# Lynch syndrome caused by a pathogenic SINE-VNTR-Alu (SVA) insertion in *MSH2* gene identified by long-read DNA sequencing

**DOI:** 10.1007/s10689-026-00588-7

**Published:** 2026-07-09

**Authors:** Jihoon E. Joo, Khalid Mahmood, Mark Clendenning, Peter Georgeson, Romy Walker, Julia Como, Fiona Phillips, Bernard J. Pope, Steven Batinovic, Natalie Diepenhorst, Julie McDonald, Toni Rice, Christophe Rosty, Mark A. Jenkins, Finlay A. Macrae, Ingrid M. Winship, Hilda High, Daniel D. Buchanan

**Affiliations:** 1 Colorectal Oncogenomics Group, Department of Clinical Pathology, Melbourne Medical School, The University of Melbourne, Parkville, VIC Australia; 2 Collaborative Centre for Genomic Cancer Medicine, Victorian Comprehensive Cancer Centre, The University of Melbourne, Parkville, VIC Australia; 3 Melbourne Bioinformatics, The University of Melbourne, Melbourne, VIC Australia; 4 Oxford Nanopore Technologies, Melbourne, VIC Australia; 5 Envoi Specialist Pathologists, Brisbane, QLD Australia; 6 University of Queensland, Brisbane, QLD Australia; 7 Centre for Epidemiology and Biostatistics, Melbourne School of Population and Global Health, The University of Melbourne, Melbourne, VIC Australia; 8 Department of Gastroenterology, Colorectal Medicine and Genetics, The Royal Melbourne Hospital, Parkville, VIC Australia; 9 Genomic Medicine and Family Cancer Clinic, The Royal Melbourne Hospital, Parkville, Melbourne, VIC Australia; 10 Department of Medicine, The University of Melbourne, Parkville, VIC Australia; 11 Sydney Cancer Genetics, Sydney, NSW Australia

**Keywords:** Lynch syndrome, DNA mismatch repair genes, Long-read sequencing, Oxford Nanopore Technologies, SINE-VNTR-Alu (SVA) insertion, Retrotransposon insertion, Structural variant

## Abstract

Lynch syndrome, the most common hereditary cancer syndrome, is caused by germline pathogenic variants in DNA mismatch repair (MMR) genes. Identifying complex or structural MMR gene pathogenic variants can be challenging with short-read sequencing resulting in patients with unexplained MMR-deficient tumours. In this study, we report multiple members of a family who developed MSH2-deficient tumours where clinical multi-gene panel testing of the DNA MMR genes using short-read sequencing did not find a germline pathogenic variant. Oxford Nanopore Technologies adaptive sampling (ONT-AS) long-read sequencing, targeting 104 hereditary cancer genes, identified a shared ~ 3.2 kb SINE-VNTR-Alu (SVA) family F retrotransposon insertion within exon 12 of *MSH2* in both the proband and her father. Segregation of this *MSH2* SVA insertion by targeted PCR confirmed three additional family members as carriers, including a paternal uncle with three colorectal cancers and an MSH2-deficient sebaceous adenoma. Three of the five heterozygous carriers were cancer affected with at least one MSH2-deficient tumour each. This study demonstrates that long-read sequencing can identify structural variants that are missed by current short-read sequencing multi-gene panel testing, improving the diagnosis of Lynch syndrome. These findings support incorporating long-read sequencing into routine diagnostic workflows for patients with suspected Lynch syndrome following a negative germline test.

**Supplementary Information:**

The online version contains supplementary material available at 10.1007/s10689-026-00588-7.

## Introduction

Lynch syndrome is the most common hereditary cancer syndrome affecting ~ 1 in 280 people [1], resulting from germline pathogenic variants in the DNA mismatch repair (MMR) genes *MLH1*, *MSH2*, *MSH6*, *PMS2* and 3’ deletions in *EPCAM*. Lynch syndrome predisposes people to a high-risk of colorectal cancer (CRC) and endometrial cancer (EC) among other cancers where the tumour phenotype is characterised by high levels of microsatellite instability (MSI-high) and loss of MMR protein expression (MMR-deficiency). In addition to Lynch syndrome, somatic causes of tumour MMR-deficiency exist, including *MLH1* gene promoter hypermethylation and biallelic somatic MMR mutations, but not all people with a MMR-deficient tumour are found to result from one of these three mechanisms with the current clinical testing approaches [2, 3].

One hypothesis related to these remaining Lynch-like or suspected Lynch syndrome cases is the “missed” diagnosis of Lynch syndrome due to rare structural variants and complex rearrangements, retrotransposon element insertions or intronic variants that occur in or near regions of highly repetitive DNA sequence or in regulatory regions [4–12] that are difficult to identify or not captured with standard short-read DNA sequencing. In this study, we utilised an analytical approach combining long-read sequencing with tumour mutation profiling in a family with multiple cancer-affected individuals that was classified with a suspected Lynch syndrome diagnosis following standard clinical multi-gene panel testing using short-read sequencing.

## Methods

### Study family

The proband (P-001), a woman of White European ancestry, was diagnosed with a stage III CRC at age 40 years; the tumour demonstrated loss of MSH2 and MSH6 protein expression and retained MLH1 and PMS2 protein expression by immunohistochemistry. Clinical germline multi-gene panel testing of hereditary CRC and polyposis genes (including *APC, MLH1, MSH2, MSH6, PMS2, MUTYH*, *NTHL1*) did not identify any pathogenic/likely pathogenic variants or variants of uncertain significance in the *MSH2* gene or in the other hereditary cancer genes tested. The proband’s father (P-003) was diagnosed with oesophageal cancer (adenocarcinoma histological type) at age 63 where the tumour demonstrated loss of MSH2 and MSH6 protein expression by immunohistochemistry, and prostate cancer at age 73 (MMR status unknown). A paternal uncle developed multiple primary CRCs at 34, 50 and 53 years with the later tumour showing loss of MSH2/MSH6 expression, and a sebaceous adenoma at 69 years also showing loss of MSH2/MSH6 expression. In addition to the proband, the proband’s father and paternal uncle also had undergone clinical germline multigene panel testing by independent clinical laboratories and without any pathogenic/likely pathogenic variants in the *MSH2* or other hereditary cancer genes identified. The proband was one of non-identical quadruplet siblings, none of whom had been diagnosed with cancer. The family was recruited to the ANGELS study (The University of Melbourne Human Research Ethics Committee approved HREC# 1750748) [3] with their cancer history and the testing completed shown in Table 1.

**Table 1:** Family members who were study participants and their cancer history

Participant	Relationship to the proband	Cancer diagnosis (age)	Mismatch repair status	Age of last contact
P-001	Proband	Colorectal cancer (40y)	MSH2/MSH6-loss	42y
P-003	Father	Oesophagus (63y) Prostate (73y)	MSH2/MSH6-loss (Oesophagus)	74y
P-009	Paternal uncle	Colorectal cancer (34y, 50y, 53y) Sebaceous adenoma (69y)	MSH2/MSH6-loss (Colorectal cancer at 53y and sebaceous adenoma)	76y
P-010	Postzygotic sibling (quadruplet)	No diagnosis	na	42y
P-011	Postzygotic sibling (quadruplet)	No diagnosis	na	42y
P-014	Postzygotic sibling (quadruplet)	No diagnosis	na	42y
P-012	Older sibling	No diagnosis	na	47y
P-013	Paternal aunt	No diagnosis	na	68y

na= not applicable

### Oxford Nanopore Technologies long-read DNA sequencing

Genomic DNA from peripheral blood samples from eight family members (P-001, P-003, P-009, P-010, P-011, P-012, P-013 and P-014, Table 1) was isolated using the QIAamp DNA Blood Midi kit (Qiagen, Germany). Oxford Nanopore Technologies (ONT) adaptive sampling was performed on blood-derived DNA from the proband (P-001) and her father (P-003), using a custom panel targeting 104 hereditary cancer genes (detailed in Supplementary Table 1), including the DNA MMR genes. Briefly, 2 µg of genomic DNA was mechanically fragmented using a Covaris g-TUBE (PerkinElmer, IL). Fragmented DNA underwent repair and end-preparation using the NEBNext FFPE DNA Repair Kit and Ultra II End Repair Kit (New England Biolabs, MA). Samples were barcoded using the Native Barcoding Sequencing Kit and sequenced on a PromethION 2 platform (Oxford Nanopore Technologies, UK). The library preparation and sequencing were performed at the Peter MacCallum Cancer Centre Molecular Genomics Core (Melbourne, Australia). Base calling was conducted using *dorado* v1.3.1 with the super-accurate (“sup”) model, with detection of modified bases including 5-methylcytosine (5mC) and 5-hydroxymethylcytosine (5hmC). Single nucleotide variants (SNVs) and structural variants (SVs) were identified using the EPI2ME “wf-human-variation” workflow with default parameters on Spartan University of Melbourne high-performance computing cluster.

### Whole exome short-read sequencing

Whole exome sequencing (WES) was performed on tumour- and matched blood-derived DNA from the proband. Genomic DNA was isolated from tumour-rich regions, and macrodissected from formalin-fixed paraffin-embedded (FFPE) specimens from the proband’s CRC. WES was performed using the SureSelect Clinical Research Exome v2 (Agilent Technologies, CA). The detailed methods of WES and calculating tumour mutational signature, tumour mutational burden (TMB; hypermutated when ≥ 10mutations/Mb) and tumour microsatellite instability using MANTIS, MSIsensor, and MSIseq tools has been described previously [3, 13, 14]. Tumour MMR-deficiency was considered present when high levels of ID2 + ID7 signature (≥ 0.605), MANTIS (≥ 0.2447). MSIsensor (≥ 5.41) and MSIseq (≥ 2.987) were observed. Loss-Of-Heterozygosity (LOH) was assessed using LOHdeTerminator (https://github.com/supernifty/LOHdeTerminator) as previously described [15].

### Targeted PCR

A targeted PCR assay was performed on blood-derived DNA from eight consented family members to test for the presence of the *MSH2* variant. Two sets of primers (Integrated DNA Technology, Singapore), one spanning across the 5′ *breakpoint* of the candidate structural variant (variant allele) and one spanning *MSH2* exon 8 (positive control), were designed and amplified using Platinum II Taq Hot-Start DNA Polymerase (Thermo Fisher Scientific, MA). The amplicons from the same individuals were pooled and the presence of the candidate *MSH2* structural variant was confirmed by gel electrophoresis. The primer sequences are shown in Supplementary Table 2.

## Results

**Fig. 1:**
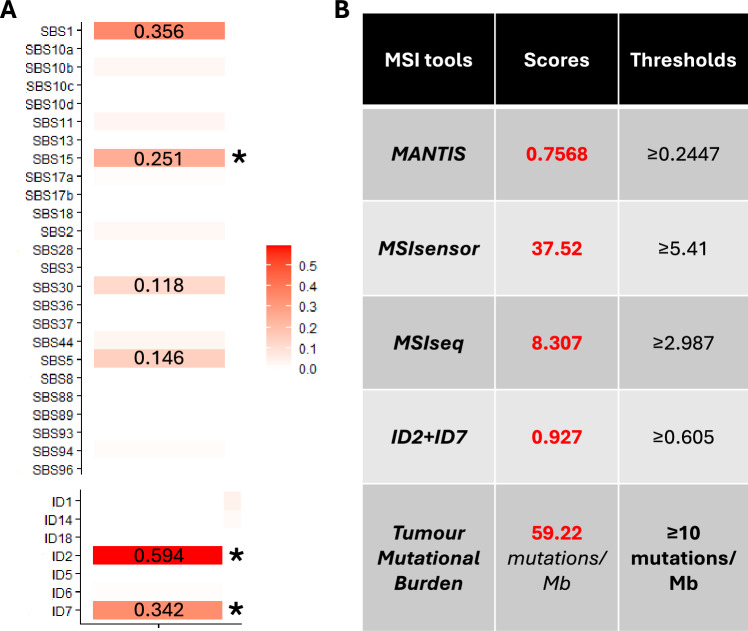
**A** The single base substitution (SBS) and indel (ID) tumour mutational signature profile of the MSH2/MSH6-deficient CRC from the proband. * denotes mismatch repair deficiency (MMR-deficiency) related mutational signatures. **B** Bioinformatically derived microsatellite instability scores using MANTIS, MSIsensor, MSIseq, ID2 + ID7 and tumour mutational burden. Red text denotes scores above our internally-derived thresholds for suggesting microsatellite instability and MMR-deficiency (see [14]).

The proband’s (P-001) right-sided colon cancer (pT3N1b) at 40 years of age showed loss of MSH2 and MSH6 protein expression by immunohistochemistry. WES of the CRC tumour and matched blood-derived DNA showed a high tumour mutational burden (59.22 mutations/Mb), a predominance of COSMIC mutational signatures SBS15 (25.1%), and both ID2 (59.4%) and ID7 (34.2%) (Fig. 1A) and high levels of microsatellite instability determined by MANTIS, MSIsensor, and MSIseq (Fig. 1B), all consistent with tumour MMR-deficiency. The tumour harboured a single somatic *MSH2* c.1477C > T (p.G493*) pathogenic variant. No LOH of the *MSH2* locus was identified by tumour WES. The proband had two additional relatives diagnosed with one or more MSH2/MSH6-deficient cancers. Collectively, these features were consistent with a suspicion of Lynch syndrome related to a *MSH2* gene defect rather than biallelic somatic MMR mutation deficiency or a false positive MMR immunohistochemistry result.

**Fig. 2:**
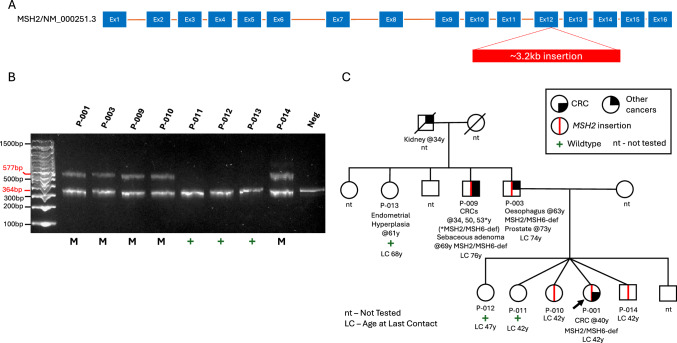
**A.** schematic diagram showing the SVA insertion (~ 3.2 kb) in *MSH2* exon 12 identified by Oxford Nanopore Technologies long-read sequencing analysis in the proband and the father with unexplained MSH2/MSH6-deficient CRC and oesophageal cancer, respectively. **B.** gel electrophoresis of PCR product targeting a part of the SVA insertion (577 bp) and *MSH2* exon 8 (364 bp) used as positive control. M = the variant identified; green +  = wildtype; Neg = unrelated individual used as a negative control. **C.** pedigree of the Lynch syndrome family identified with the *MSH2 exon 12* SVA insertion. *MMR Immunohistochemistry only available from CRC diagnosed at 53y and showed MSH2/MSH6 protein expression loss. LC – Age at Last Contact. nt – not tested

Clinical germline multigene panel testing did not identify a pathogenic or likely pathogenic variant in *MSH2* or in the other known CRC predisposition genes tested. No additional germline pathogenic variants were identified from WES testing in P-001. Oxford Nanopore Technologies adaptive sequencing (ONT-AS) on blood-derived DNA from the proband (P-001) and her father (P-003) identified a shared ~3.2 kb insertion within exon 12 of *MSH2* (NM_000251.3 c.1972_1973ins, p.?) in both individuals (Fig. 2A and Supplementary Fig. 1). The inserted sequence showed homology to a SINE-VNTR-Alu (SVA) family F retrotransposon (Supplementary Information). No *MSH2* promoter hypermethylation or a deletion of 3’ end of the *EPCAM* gene was identified by ONT sequencing of blood-derived DNA samples of the proband and the father (Supplementary Fig. 2).

A targeted PCR assay spanning the *breakpoint* between exon 12 and the SVA insertion was used to assess segregation of the *MSH2* 3.2 kb insertion within the family. In addition to the proband, the SVA insertion was detected in two of the five siblings with both from the postzygotic quadruplets, although neither had developed cancer by age 42. A paternal uncle was also identified to carry the SVA insertion. He developed three CRCs, the later with loss of MSH2/MSH6 expression, and a sebaceous adenoma at 69yrs also with loss of MSH2/MSH6 expression (Table 1; Fig. 2B, C).

## Discussion

In this study, we identified a germline SVA family F retrotransposon insertion of ~3.2 kb within exon 12 of *MSH2* using ONT long-read adaptive sampling sequencing that segregated with five family members, three of them cancer-affected including multiple CRCs, oesophageal cancer and a sebaceous adenoma that each demonstrated loss of MSH2/MSH6 protein expression. Clinical multi-gene panel testing of the proband, proband’s father and paternal uncle found no evidence of the SVA insertion and was not detected by the variant caller from our WES analysis. This finding highlights the diagnostic utility of long-read sequencing to detect rare complex genetic variants in people who have a high suspicion of Lynch syndrome.

Contemporary clinical testing for Lynch syndrome relies on multigene panel testing using short-read sequencing technologies. It is recognised that short-read sequencing has limitations for detection of structural variants, complex rearrangements, intronic variants that occur in or near regions of highly repetitive DNA sequence or in regulatory regions [16]. The SVA insertion in this family was not detected by clinical multigene panel testing or called from our WES analysis (although there were reads related to the insertion present (Supplementary Fig. 1)). Employing bioinformatic tools specifically designed to detect transposable elements such as xTea (x-Transposable Element Analyzer) [17] or TraFiC (Transposon Finder in Cancer) [18] could improve detection of transposable element insertions although such tools would not recover the complete insertion sequence, which would require long-read sequencing as applied in this study. We have previously shown the value of combining tumour testing and segregation analysis in multiple family members to facilitate detection of cryptic germline pathogenic variants in the *MSH2* gene [8, 19]. Combining tumour and polyp profiling with long-read sequencing has the potential to improve the diagnostic yield for detecting cryptic germline pathogenic variants in hereditary CRC and polyposis susceptibility genes.

Sjursen et al. reported that there are > 40 cases of structural variants including retrotransposon insertions of various sizes ranging from ~100 bp to ~6 kb in the DNA MMR genes [4, 5, 9–11, 20–24]. A previous report from the USA described an SVA insertion in *MSH2* exon 12 [7], which is ostensibly identical to the SVA family F found in our study. The partial sequence (~660 bp) of the SVA insertion provided in the study showed > 91% homology to the ~3.2 kb SVA insertion identified in our study that was discovered by the long-read sequencing, suggesting that the two insertions may be identical. No ethnic or geographic background of the USA family was available, therefore, we could not assess the relatedness of the USA and the Australian families. A large insertion at this location has also been reported in ClinVar (https://www.ncbi.nlm.nih.gov/clinvar/variation/1072831/; last accessed on 9th June, 2026). The partial ~125 bp sequence showed > 98% homology to the 3.2 kb SVA insertion, suggesting these three reports of an SVA insertion in *MSH2* exon12 are the same variant.

It has been suggested that retrotransposon element insertions are a potentially underdiagnosed cause of Lynch syndrome given their difficulty in detection using conventional methodologies [10]. The family presented in this study and the accumulating evidence support the incorporation of long-read sequencing into the diagnostic workflow for people with a high suspicion of Lynch syndrome following a negative germline multigene panel test. Further incorporation of tumour profiling can guide germline investigations and remove doubt related to a false positive MMR-deficiency immunohistochemistry result. In the future, long-read sequencing should be considered as a standard component of the diagnostic workup for patients with a suspicion of Lynch syndrome with the potential for broader application to improve the diagnostic yield for other hereditary CRC and polyposis genes following standard short-read germline testing.

## Supplementary Information

Below is the link to the electronic supplementary material.

Supplementary file1 (HTML 75 KB)

Supplementary file2 (PDF 781 KB)

Supplementary file3 (XLSX 15 KB)

## Data Availability

No datasets were generated or analysed during the current study.
