# Supplementary material for: Lynch syndrome caused by a pathogenic SINE-VNTR-Alu (SVA) insertion in MSH2 gene identified by long-read DNA sequencing
Source: Fam Cancer. 2026 Jul 9;25(3):75. doi: 10.1007/s10689-026-00588-7 (PMC13350196; doi:10.1007/s10689-026-00588-7)
Supplement: Supplementary file 1 — Supplementary file1 (HTML 75 KB) [file 10689_2026_588_MOESM1_ESM.html]

[ close all hsps ]
[ open all hsps ]

```
                                          position in query-                           -position in repeat-
          %    %    %    query                               C matching repeat         (left)  end   begin  linkage
+ score  div. del. ins.  sequence         begin  end  (left) + repeat   class/family   begin   end   (left) id/graphic
```

```
+   37    2.6  0.0  0.0  UnnamedSequence     22    60 (3147) + (T)n     Simple_repeat       1     39    (0)   1
```

```
ANNOTATION EVIDENCE:
   37   2.61 0.00 0.00  UnnamedSequence     22    60   3147 + (T)n     Simple_repeat       1     39      0    
37 2.61 0.00 0.00 UnnamedSequence 22 60 (3147) (T)n#Simple_repeat 1 39 (0) c_b1s251i0

  UnnamedSequen         22 TTTTTTTTTTTTTTTTTTTTTTTTTTTTTTTATTTTTTT 60
                                                          v       
  (T)n#Simple_r          1 TTTTTTTTTTTTTTTTTTTTTTTTTTTTTTTTTTTTTTT 39

Matrix = Unknown
Transitions / transversions = 0.00 (0/1)
Gap_init rate = 0.00 (0 / 38), avg. gap size = 0.0 (0 / 0)
```

```
+ 6239    8.0  2.1  8.8  UnnamedSequence     68  2914  (293) C SVA_F    Retroposon/SVA   (13)   1362    392   2
```

```
ANNOTATION EVIDENCE:
 6239   3.75 0.30 5.53  UnnamedSequence     68  1057   2150 C SVA_F    Retroposon/SVA    422   1362     13    
6239 3.75 0.30 5.53 UnnamedSequence 68 1057 (2150) C SVA_F#Retroposon/SVA (13) 1362 422 m_b1s502i0

  UnnamedSequen         68 TTTTTTTATTGATAATTCTTGGGTGTTTCTCACAGAGGGGGATTTGGCAG 117
                                                                             
C SVA_F#Retropo       1362 TTTTTTTATTGATAATTCTTGGGTGTTTCTCACAGAGGGGGATTTGGCAG 1313

  UnnamedSequen        118 GGTCATGGGACAATAGTGGAGGGAAGGTCAGCAGATAAACAAGTGAACAA 167
                                                                             
C SVA_F#Retropo       1312 GGTCATGGGACAATAGTGGAGGGAAGGTCAGCAGATAAACAAGTGAACAA 1263

  UnnamedSequen        168 AGGTCTCTGGTTTTCCTAGGCAGAGGACCCTGCGGCCTTCCGCAGATGTT 217
                                                                        -    
C SVA_F#Retropo       1262 AGGTCTCTGGTTTTCCTAGGCAGAGGACCCTGCGGCCTTCCGCAG-TGTT 1214

  UnnamedSequen        218 TCTGTCCCTGATTACTTGAGATTAGGGATTGGTGATGACTCTTAACGAGC 267
                            v                                                
C SVA_F#Retropo       1213 TGTGTCCCTGATTACTTGAGATTAGGGATTGGTGATGACTCTTAACGAGC 1164

  UnnamedSequen        268 ATGCTGCCTTCAAGCATCTGTTTGACAAATCACATCTTGCACCGCCCTTA 317
                                                  i     v                    
C SVA_F#Retropo       1163 ATGCTGCCTTCAAGCATCTGTTTAACAAAGCACATCTTGCACCGCCCTTA 1114

  UnnamedSequen        318 ATCCATTTAACCCTGAGTGGACACAGCACATGTTTCAGAGAGCACAGGGT 367
                                                                             
C SVA_F#Retropo       1113 ATCCATTTAACCCTGAGTGGACACAGCACATGTTTCAGAGAGCACAGGGT 1064

  UnnamedSequen        368 TGGGGGTAAGGTCACAGATCAACAGGATCCCAAGGCAGAGGAATTTTTCT 417
                                                                             
C SVA_F#Retropo       1063 TGGGGGTAAGGTCACAGATCAACAGGATCCCAAGGCAGAGGAATTTTTCT 1014

  UnnamedSequen        418 TAGTGCAGAACAAAATGAAAAGTCTCCCATGTCTACTTCTTTCTACACAG 467
                                                                             
C SVA_F#Retropo       1013 TAGTGCAGAACAAAATGAAAAGTCTCCCATGTCTACTTCTTTCTACACAG 964

  UnnamedSequen        468 ACACGGCAACCATCCGATTTCTCAATCTTTTCCCCACCTTTCCCGCCTTT 517
                                                                             
C SVA_F#Retropo        963 ACACGGCAACCATCCGATTTCTCAATCTTTTCCCCACCTTTCCCGCCTTT 914

  UnnamedSequen        518 CTATTCCACAAAGCCGCCATTGTCATCCTGGCCCGTTCTCAATGAGCTGT 567
                                                                             
C SVA_F#Retropo        913 CTATTCCACAAAGCCGCCATTGTCATCCTGGCCCGTTCTCAATGAGCTGT 864

  UnnamedSequen        568 TGGGCACACCTCCCAGACGGGGTGGTGGCCGGGCAGAGGGGCTCCTCACT 617
                                         i       i                           
C SVA_F#Retropo        863 TGGGCACACCTCCCGGACGGGGCGGTGGCCGGGCAGAGGGGCTCCTCACT 814

  UnnamedSequen        618 TCCCAGTAGGGGCGGCCGGGCAGAGGCGCCCCTCACCTCCCGGACGGGGC 667
                                                                             
C SVA_F#Retropo        813 TCCCAGTAGGGGCGGCCGGGCAGAGGCGCCCCTCACCTCCCGGACGGGGC 764

  UnnamedSequen        668 GGCTGGCCGGGCAGGGGGGCTGACCCCCCCACCTCCCTCCTGGACGGGGC 717
                                       -                     -     i         
C SVA_F#Retropo        763 GGCTGGCCGGGC-GGGGGGCTGACCCCCCCACCT-CCTCCCGGACGGGGC 716

  UnnamedSequen        718 GGCTGGCCGGGCGGGGGGCTGACCCCCCAACCTCCCTCCCGGACGGGGCG 767
                                                       v ----                
C SVA_F#Retropo        715 GGCTGGCCGGGCGGGGGGCTGACCCCCCCA----CCTCCCGGACGGGGCG 670

  UnnamedSequen        768 GCTGGCCGGGCGGGGG-GCTGACCCCCCCACCTCCCTCCCGGACGGGGCG 816
                                        i  -   -----  i  ---- ?             -
C SVA_F#Retropo        669 GCTGGCCGGGCGGAGGCGCT-----CCTCA----CNTCCCGGACGGGGC- 630

  UnnamedSequen        817 GCTGGCCGGGCAGAGGGGCTCCTCACTTCCCAGT--AGGGGCGGCCGGGC 864
                           ---        i    v         ?    i v--i?            
C SVA_F#Retropo        629 ---GGCCGGGCGGAGGCGCTCCTCACNTCCCGGACGGNGGGCGGCCGGGC 583

  UnnamedSequen        865 AGAGGCGCCCCTCACCTCCCGGACGGGGCGGCTGGCCGGACGGGGGGCCG 914
                           i   i   i                       -      i   i  --  
C SVA_F#Retropo        582 GGAGACGCTCCTCACCTCCCGGACGGGGCGGC-GGCCGGGCGGAGG--CG 536

  UnnamedSequen        915 ACCCCCCCACCTCCCTCCCGGACGGGGCGGCTGGCCGGGCGGGGGGCCGA 964
                           -- i  i  ----      i        ----        i i   ----
C SVA_F#Retropo        535 --CTCCTCA----CCTCCCAGACGGGGC----GGCCGGGCAGAGGG---- 500

  UnnamedSequen        965 CCCCCCCACCTCCCTCCCGGACGGGGCGGCTGGCCGGGCGGGGGGCCGAC 1014
                           v i  i  ---- ?    i    iv -   -        i i  --  --
C SVA_F#Retropo        499 GCTCCTCA----CNTCCCAGACGATG-GGC-GGCCGGGCAGAGG--CG-- 460

  UnnamedSequen       1015 CCCCCCACCTCCCTCCCGGACGGGGCGGCTGGCCGGGCAGAGG 1057
                            i  i  ----      i           -             
C SVA_F#Retropo        459 CTCCTCA----CCTCCCAGACGGGGCGGC-GGCCGGGCAGAGG 422

Matrix = 20p53g.matrix
Kimura (with divCpGMod) = 3.25
Transitions / transversions = 4.00 (28/7)
Gap_init rate = 0.05 (54 / 989), avg. gap size = 1.02 (55 / 54)

 1059   9.32 2.27 13.76  UnnamedSequence    651  1135   2072 C SVA_F    Retroposon/SVA    422    857    518    
1059 9.32 2.27 13.76 UnnamedSequence 651 1135 (2072) C SVA_F#Retroposon/SVA (518) 857 422 m_b1s502i1

  UnnamedSequen        651 CACCTCCCGGACGGGGCGGCTGGCCGGGCAGGGGGGCTGACCCCCCCACC 700
                                              -           i      -----  i   i
C SVA_F#Retropo        857 CACCTCCCGGACGGGGCGG-TGGCCGGGCAGAGGGGCT-----CCTCACT 814

  UnnamedSequen        701 TCCCTCCTGGACGGGGCGGCTGGCCGGGCGGGGGGCTGACCCCCCAACCT 750
                               ----i vv        ----     i i  v---i    i  ----
C SVA_F#Retropo        813 TCCC----AGTAGGGGCGGC----CGGGCAGAGGC---GCCCCTCA---- 779

  UnnamedSequen        751 CCCTCCCGGACGGGGCGGCTGGCCGGGCGGGGGGCTGACCCCCCCACCTC 800
                           -                                                -
C SVA_F#Retropo        778 -CCTCCCGGACGGGGCGGCTGGCCGGGCGGGGGGCTGACCCCCCCACCT- 731

  UnnamedSequen        801 CCTCCCGGACGGGGCGGCTGGCCGGGCAGAGGGGCT-----CCTCACTTC 845
                                                      - i      -----  i   i  
C SVA_F#Retropo        730 CCTCCCGGACGGGGCGGCTGGCCGGGC-GGGGGGCTGACCCCCCCACCTC 682

  UnnamedSequen        846 CCAGTAGGGGC----GGCCGGGCAGAGGCGCCCCTCACCTCCCGGACGGG 891
                             i vv     ----        i       i      ?           
C SVA_F#Retropo        681 CCGGACGGGGCGGCTGGCCGGGCGGAGGCGCTCCTCACNTCCCGGACGGG 632

  UnnamedSequen        892 GCGGCTGGCCGGACGGGGGGCCGACCCCCCCACCTCCCTCCCGGACGGGG 941
                             ----      i   i  --  -- i  i  ---- ?          ? 
C SVA_F#Retropo        631 GC----GGCCGGGCGGAGG--CG--CTCCTCA----CNTCCCGGACGGNG 594

  UnnamedSequen        942 CGGCTGGCCGGGCGGGGGGCCGACCCCCCCACCTCCCTCCCGGACGGGGC 991
                           -   -          i i--  -- i  i  ----               
C SVA_F#Retropo        593 -GGC-GGCCGGGCGGAGA--CG--CTCCTCA----CCTCCCGGACGGGGC 554

  UnnamedSequen        992 GGCTGGCCGGGCGGGGGGCCGACCCCCCCACCTCCCTCCCGGACGGGGCG 1041
                              -          i  --  -- i  i  ----      i        -
C SVA_F#Retropo        553 GGC-GGCCGGGCGGAGG--CG--CTCCTCA----CCTCCCAGACGGGGC- 514

  UnnamedSequen       1042 GCTGGCCGGGCAGAGGGGCTCCTCACTTCCCAGT--AGGGGCGGCCGGGC 1089
                           ---                       ?      v-- v            
C SVA_F#Retropo        513 ---GGCCGGGCAGAGGGGCTCCTCACNTCCCAGACGATGGGCGGCCGGGC 467

  UnnamedSequen       1090 AGAGGCGCCCCTCACCTCCCGGACAGGGCGGCTGGCTGGGCGGGGG 1135
                                   i           i   i       -   i    i i  
C SVA_F#Retropo        466 AGAGGCGCTCCTCACCTCCCAGACGGGGCGGC-GGCCGGGCAGAGG 422

Matrix = 20p53g.matrix
Kimura (with divCpGMod) = 8.81
Transitions / transversions = 4.57 (32/7)
Gap_init rate = 0.13 (63 / 484), avg. gap size = 1.13 (71 / 63)

  735  11.94 9.15 9.91  UnnamedSequence    752  1188   2019 C SVA_E    Retroposon/SVA    429    862    520    
735 11.94 9.15 9.91 UnnamedSequence 752 1188 (2019) C SVA_E#Retroposon/SVA (520) 862 429 m_b1s502i2

  UnnamedSequen        752 CCTCCCGGACGGGGCGGCTGGCCGGGCGG-GGGGCTGACCCCCCCACCTC 800
                                            -         i -      -----  i   i  
C SVA_E#Retropo        862 CCTCCCGGACGGGGCGG-TGGCCGGGCAGAGGGGCT-----CCTCACTTC 819

  UnnamedSequen        801 CCTCCCGGACGGGGCGGCTGGCCGGGCAGAGGGGCTCCTCACTTCCCAGT 850
                             ----i vv        ----          v  i      i    i v
C SVA_E#Retropo        818 CC----AGTAGGGGCGGC----CGGGCAGAGGCGCCCCTCACCTCCCGGA 777

  UnnamedSequen        851 AGGGGCGGC----CGGGCAGAGG--CG--CCCCTCACCTCC---CGGACG 889
                           v        ----     i i  --i --    i       ---      
C SVA_E#Retropo        776 CGGGGCGGCTGGCCGGGCGGGGGGCTGACCCCCCCACCTCCTCCCGGACG 727

  UnnamedSequen        890 GGGCGGCTGGCCGGACGGGGGGCCGACCCCCCCACCTCCCTCCCGGACGG 939
                                         i        i                ----      
C SVA_E#Retropo        726 GGGCGGCTGGCCGGGCGGGGGGCTGACCCCCCCACCTCCC----GGACGG 681

  UnnamedSequen        940 GGCGGCTGGCCGGGCGGGGGGCCGACCCCCCCACCTCCCTCCCGGACGGG 989
                                            i  --  -- i  i        ----       
C SVA_E#Retropo        680 GGCGGCTGGCCGGGCGGAGG--CG--CTCCTCACCTCCC----GGACGGG 639

  UnnamedSequen        990 GCGGCTGG--------------------CCGGGCGGGGGGCCGACCCCCC 1019
                                ?  --------------------    i  ??    - i  vvv 
C SVA_E#Retropo        638 GCGGCNGGGCGGAGGCGCTCCCCACNTCCCGGACGNNGGGC-GGCCGGGC 590

  UnnamedSequen       1020 -------CACCTC-CCTCCCGGACGGGGCGGCTGGCCGGGCAGAGGGGCT 1061
                           ------- v    - i    i           -             v   
C SVA_E#Retropo        589 AGAGGCGCTCCTCACTTCCCAGACGGGGCGGC-GGCCGGGCAGAGGCGCT 541

  UnnamedSequen       1062 CCTCACTTCCCAGTAGGGGCGGCCGGGCAGAGGCGCCCCTCACCTCCCGG 1111
                                 i    i vv                  v  i      ?      
C SVA_E#Retropo        540 CCTCACCTCCCGGACGGGGCGGCCGGGCAGAGGGGCTCCTCACNTCCCGG 491

  UnnamedSequen       1112 ACAGGGCGGCTGGCTGGGCGGGGGGGCTGACCCCCCCCCCACCTCCCTCC 1161
                             -- ii  vi   i    i i  v   --------  i  ----     
C SVA_E#Retropo        490 AC--GATGGGCGGCCGGGCAGAGGCGCT--------CCTCA----CCTCC 455

  UnnamedSequen       1162 CGGACGGGGCGGCTGGCCGGGCGGGGG 1188
                                        -        i i  
C SVA_E#Retropo        454 CGGACGGGGCGGC-GGCCGGGCAGAGG 429

Matrix = 20p53g.matrix
Kimura (with divCpGMod) = 11.89
Transitions / transversions = 2.13 (32/15)
Gap_init rate = 0.12 (51 / 436), avg. gap size = 1.63 (83 / 51)

 1632   7.07 1.26 10.57  UnnamedSequence    801  1275   1932 C SVA_F    Retroposon/SVA    421    855    520    
1632 7.07 1.26 10.57 UnnamedSequence 801 1275 (1932) C SVA_F#Retroposon/SVA (520) 855 421 m_b1s502i3

  UnnamedSequen        801 CCTCCCGGACGGGGCGGCTGGCCGGGCAGAGGGGCTCCTCACTTCCCAGT 850
                                            -                                
C SVA_F#Retropo        855 CCTCCCGGACGGGGCGG-TGGCCGGGCAGAGGGGCTCCTCACTTCCCAGT 807

  UnnamedSequen        851 AGGGGCGGCCGGGCAGAGGCGCCCCTCACCTCCCGGACGGGGCGGCTGGC 900
                                                                             
C SVA_F#Retropo        806 AGGGGCGGCCGGGCAGAGGCGCCCCTCACCTCCCGGACGGGGCGGCTGGC 757

  UnnamedSequen        901 CGGACGGGGGGCCGACCCCCCCACCTCCCTCCCGGACGGGGCGGCTGGCC 950
                              i        i             -                       
C SVA_F#Retropo        756 CGGGCGGGGGGCTGACCCCCCCACCT-CCTCCCGGACGGGGCGGCTGGCC 708

  UnnamedSequen        951 GGGCGGGGGGCCGACCCCCCCACCTCCCTCCCGGACGGGGCGGCTGGCCG 1000
                                      i          ----                        
C SVA_F#Retropo        707 GGGCGGGGGGCTGACCCCCCCA----CCTCCCGGACGGGGCGGCTGGCCG 662

  UnnamedSequen       1001 GGCGGGGGGCCGACCCCCCCACCTCCCTCCCGGACGGGGCGGCTGGCCGG 1050
                                i  --  -- i  i  ---- ?             ----      
C SVA_F#Retropo        661 GGCGGAGG--CG--CTCCTCA----CNTCCCGGACGGGGC----GGCCGG 624

  UnnamedSequen       1051 GCAGAGGGGCTCCTCACTTCCCAGT--AGGGGCGGCCGGGCAGAGGCGCC 1098
                             i    v         ?    i v--i?            i   i   i
C SVA_F#Retropo        623 GCGGAGGCGCTCCTCACNTCCCGGACGGNGGGCGGCCGGGCGGAGACGCT 574

  UnnamedSequen       1099 CCTCACCTCCCGGACAGGGCGGCTGGCTGGGCGGGGGGGCTGACCCCCCC 1148
                                          i       -   i      i  v   -------- 
C SVA_F#Retropo        573 CCTCACCTCCCGGACGGGGCGGC-GGCCGGGCGGAGGCGCT--------C 533

  UnnamedSequen       1149 CCCACCTCCCTCCCGGACGGGGCGGCTGGCCGGGC-GGGGGGCTGACCCC 1197
                            i  ----      i        ----        - i      ----- 
C SVA_F#Retropo        532 CTCA----CCTCCCAGACGGGGC----GGCCGGGCAGAGGGGCT-----C 496

  UnnamedSequen       1198 CCCACCTCCCTCCCGGACGGGGCGGCTGGCCGGGCAGAGGGGCTCCTCAC 1247
                            i  ---- ?    i    iv -   -             v         
C SVA_F#Retropo        495 CTCA----CNTCCCAGACGATG-GGC-GGCCGGGCAGAGGCGCTCCTCAC 452

  UnnamedSequen       1248 TTCCCAGTAGG---GGCGGCCGAGCAGAGGC 1275
                           i      vv  ---        i        
C SVA_F#Retropo        451 CTCCCAGACGGGGCGGCGGCCGGGCAGAGGC 421

Matrix = 20p53g.matrix
Kimura (with divCpGMod) = 6.30
Transitions / transversions = 3.29 (23/7)
Gap_init rate = 0.10 (49 / 474), avg. gap size = 1.06 (52 / 49)

  912  11.25 2.03 15.14  UnnamedSequence    877  1368   1839 C SVA_E    Retroposon/SVA    429    864    518    
912 11.25 2.03 15.14 UnnamedSequence 877 1368 (1839) C SVA_E#Retroposon/SVA (518) 864 429 m_b1s502i4

  UnnamedSequen        877 CACCTCCCGGACGGGGCGGCTGGCCGGACGGGGGGCCGACCCCCCCACCT 926
                                              -       i i i   v ----i  i   i 
C SVA_E#Retropo        864 CACCTCCCGGACGGGGCGG-TGGCCGGGCAGAGGGGC----TCCTCACTT 820

  UnnamedSequen        927 CCCTCCCGGACGGGGCGGCTGGCCGGGCGGGGGGCCGACCCCCCCACCTC 976
                              ----i vv        ----     i i  --  --    i      
C SVA_E#Retropo        819 CCC----AGTAGGGGCGGC----CGGGCAGAGG--CG--CCCCTCACCTC 782

  UnnamedSequen        977 CCTCCCGGACGGGGCGGCTGGCCGGGCGGGGGGCCGACCCCCCCACCTCC 1026
                             ----                            i               
C SVA_E#Retropo        781 CC----GGACGGGGCGGCTGGCCGGGCGGGGGGCTGACCCCCCCACCTCC 736

  UnnamedSequen       1027 CTCCCGGACGGGGCGGCTGGCCGGGCAGAGGGGCT-----CCTCACTTCC 1071
                           -                         i -      -----  i   i   
C SVA_E#Retropo        735 -TCCCGGACGGGGCGGCTGGCCGGGCGG-GGGGCTGACCCCCCCACCTCC 688

  UnnamedSequen       1072 CAGTAGGGGCGGC----CGGGCAGAGGCGCCCCTCACCTCCCGGACAGGG 1117
                            i vv        ----     i       i               i   
C SVA_E#Retropo        687 CGGACGGGGCGGCTGGCCGGGCGGAGGCGCTCCTCACCTCCCGGACGGGG 638

  UnnamedSequen       1118 CGGCTGGCTGGGCGGGGGGGCTGACCCCCCCCCCACCTCCCTCCCGGACG 1167
                               ?  ----    i  v   --------      ?    ----     
C SVA_E#Retropo        637 CGGCNGG----GCGGAGGCGCT--------CCCCACNTCCC----GGACG 604

  UnnamedSequen       1168 GGGCGGCTGGCCGGGCGGGGG-GCTGACCCCCCCACCTCCCTCCCGGACG 1216
                           ?? -   -        i i  -   -----  i   i    ----i    
C SVA_E#Retropo        603 NNG-GGC-GGCCGGGCAGAGGCGCT-----CCTCACTTCCC----AGACG 565

  UnnamedSequen       1217 GGGCGGCTGGCCGGGCAGAGGGGCTCCTCACTTCCCAGTAGGGGCGGCCG 1266
                                  -             v         i    i vv          
C SVA_E#Retropo        564 GGGCGGC-GGCCGGGCAGAGGCGCTCCTCACCTCCCGGACGGGGCGGCCG 516

  UnnamedSequen       1267 AGCAGAGGCGCCCCTCACCTCCCGGACGGGGCGGCTGGCCGGGCAGGGGG 1316
                           i       v  i      ?         iv -   -          i  -
C SVA_E#Retropo        515 GGCAGAGGGGCTCCTCACNTCCCGGACGATG-GGC-GGCCGGGCAGAGG- 469

  UnnamedSequen       1317 GCCGACCCCCCCCCCACCTCCCTCCCGGACAGGGCGGCTGGCCGGGCGGG 1366
                           ------- v i  i  ----          i       -        i i
C SVA_E#Retropo        468 -------CGCTCCTCA----CCTCCCGGACGGGGCGGC-GGCCGGGCAGA 431

  UnnamedSequen       1367 GG 1368
                             
C SVA_E#Retropo        430 GG 429

Matrix = 20p53g.matrix
Kimura (with divCpGMod) = 10.73
Transitions / transversions = 2.92 (35/12)
Gap_init rate = 0.14 (69 / 491), avg. gap size = 1.10 (76 / 69)

 1590   7.72 0.63 10.80  UnnamedSequence   1026  1504   1703 C SVA_F    Retroposon/SVA    421    855    520    
1590 7.72 0.63 10.80 UnnamedSequence 1026 1504 (1703) C SVA_F#Retroposon/SVA (520) 855 421 m_b1s502i5

  UnnamedSequen       1026 CCTCCCGGACGGGGCGGCTGGCCGGGCAGAGGGGCTCCTCACTTCCCAGT 1075
                                            -                                
C SVA_F#Retropo        855 CCTCCCGGACGGGGCGG-TGGCCGGGCAGAGGGGCTCCTCACTTCCCAGT 807

  UnnamedSequen       1076 AGGGGCGGCCGGGCAGAGGCGCCCCTCACCTCCCGGACAGGGCGGCTGGC 1125
                                                                 i           
C SVA_F#Retropo        806 AGGGGCGGCCGGGCAGAGGCGCCCCTCACCTCCCGGACGGGGCGGCTGGC 757

  UnnamedSequen       1126 TGGGCGGGGGGGCTGACCCCCCCCCCACCTCCCTCCCGGACGGGGCGGCT 1175
                           i    -          ---           -                   
C SVA_F#Retropo        756 CGGGC-GGGGGGCTGA---CCCCCCCACCT-CCTCCCGGACGGGGCGGCT 712

  UnnamedSequen       1176 GGCCGGGCGGGGGGCTGACCCCCCCACCTCCCTCCCGGACGGGGCGGCTG 1225
                                                     ----                    
C SVA_F#Retropo        711 GGCCGGGCGGGGGGCTGACCCCCCCA----CCTCCCGGACGGGGCGGCTG 666

  UnnamedSequen       1226 GCCGGGCAGAGGGGCTCCTCACTTCCCAGTAGGGGCGGCCGAGCAGAGGC 1275
                                  i    v         ?    i vv          i  i     
C SVA_F#Retropo        665 GCCGGGCGGAGGCGCTCCTCACNTCCCGGACGGGGCGGCCGGGCGGAGGC 616

  UnnamedSequen       1276 GCCCCTCACCTCCCGGACGGGGCGGCTGGCCGGGCAGGGGGGCCGACCCC 1325
                             i      ?          ? -   -        ---  i v  -----
C SVA_F#Retropo        615 GCTCCTCACNTCCCGGACGGNG-GGC-GGCCGGGC---GGAGACG----- 576

  UnnamedSequen       1326 CCCCCCACCTCCCTCCCGGACAGGGCGGCTGGCCGGGCGGGGGGCCGACC 1375
                            i  i  ----          i       -          i  --  -- 
C SVA_F#Retropo        575 CTCCTCA----CCTCCCGGACGGGGCGGC-GGCCGGGCGGAGG--CG--C 535

  UnnamedSequen       1376 CCCCCACCTCCCTCCCGGACGGGGCGGCTGGCCGGGCGGGGGGCCGACCC 1425
                           i  i  ----      i        ----        i i   ----v i
C SVA_F#Retropo        534 TCCTCA----CCTCCCAGACGGGGC----GGCCGGGCAGAGGG----GCT 497

  UnnamedSequen       1426 CCCCACCTCCCTCCCGGACAGGGCGGCTGGCCGGGCAGAGGGGCTCCTCA 1475
                             i  ---- ?    i   -- ii  vi             v        
C SVA_F#Retropo        496 CCTCA----CNTCCCAGAC--GATGGGCGGCCGGGCAGAGGCGCTCCTCA 453

  UnnamedSequen       1476 CTTCCCAGTAGG---GGCGGCCGGGCAGAGGC 1504
                            i      vv  ---                 
C SVA_F#Retropo        452 CCTCCCAGACGGGGCGGCGGCCGGGCAGAGGC 421

Matrix = 20p53g.matrix
Kimura (with divCpGMod) = 6.55
Transitions / transversions = 2.67 (24/9)
Gap_init rate = 0.10 (48 / 478), avg. gap size = 1.04 (50 / 48)

  689  12.71 9.09 10.60  UnnamedSequence   1157  1596   1611 C SVA_F    Retroposon/SVA    422    855    520    
689 12.71 9.09 10.60 UnnamedSequence 1157 1596 (1611) C SVA_F#Retroposon/SVA (520) 855 422 m_b1s502i6

  UnnamedSequen       1157 CCTCCCGGACGGGGCGGCTGGCCGGGCGG-GGGGCTGACCCCCCCACCTC 1205
                                            -         i -      -----  i   i  
C SVA_F#Retropo        855 CCTCCCGGACGGGGCGG-TGGCCGGGCAGAGGGGCT-----CCTCACTTC 812

  UnnamedSequen       1206 CCTCCCGGACGGGGCGGCTGGCCGGGCAGAGGGGCTCCTCACTTCCCAGT 1255
                             ----i vv        ----          v  i      i    i v
C SVA_F#Retropo        811 CC----AGTAGGGGCGGC----CGGGCAGAGGCGCCCCTCACCTCCCGGA 770

  UnnamedSequen       1256 AGGGGCGGC----CGAGCAGAGG--CG--CCCCTCACCTCC---CGGACG 1294
                           v        ----  i  i i  --i --    i       ---      
C SVA_F#Retropo        769 CGGGGCGGCTGGCCGGGCGGGGGGCTGACCCCCCCACCTCCTCCCGGACG 720

  UnnamedSequen       1295 GGGCGGCTGGCCGGGCAGGGGGGCCGACCCCCCCCCCACCTCCCTCCCGG 1344
                                           -       i         v  -------      
C SVA_F#Retropo        719 GGGCGGCTGGCCGGGC-GGGGGGCTGACCCCCCCACC-------TCCCGG 678

  UnnamedSequen       1345 ACAGGGCGGCTGGCCGGGCGGGGGGCCGACCCCCCCACCTCCCTCCCGGA 1394
                             i                  i  --  -- i  i   ?    ----   
C SVA_F#Retropo        677 ACGGGGCGGCTGGCCGGGCGGAGG--CG--CTCCTCACNTCCC----GGA 636

  UnnamedSequen       1395 CGGGGCGGCTGG--------------------CCGGGCGGGGGGCCGACC 1424
                                    i  --------------------    i   ?    - i  
C SVA_F#Retropo        635 CGGGGCGGCCGGGCGGAGGCGCTCCTCACNTCCCGGACGGNGGGC-GGCC 587

  UnnamedSequen       1425 C-------CCCCACCTC-CCTCCCGGACAGGGCGGCTGGCCGGGCAGAGG 1466
                           v-------v v v    -          i       -        i    
C SVA_F#Retropo        586 GGGCGGAGACGCTCCTCACCTCCCGGACGGGGCGGC-GGCCGGGCGGAGG 538

  UnnamedSequen       1467 GGCTCCTCACTTCCCAGTAGGGGCGGCCGGGCAGAGGCGCCCCTCACCTC 1516
                           v         i      vv                  v  i      ?  
C SVA_F#Retropo        537 CGCTCCTCACCTCCCAGACGGGGCGGCCGGGCAGAGGGGCTCCTCACNTC 488

  UnnamedSequen       1517 CCGGACAGGGCGGCTGGCTGGGCGGGGGGGCTGACCCCCCCCAACCTCCC 1566
                             i   -- ii  vi   i    i i  v   ------  i  -----  
C SVA_F#Retropo        487 CCAGAC--GATGGGCGGCCGGGCAGAGGCGCT------CCTCA-----CC 451

  UnnamedSequen       1567 TCCCAGACGGGGCGGCTGGCCGGGCGGGGG 1596
                                           -        i i  
C SVA_F#Retropo        450 TCCCAGACGGGGCGGC-GGCCGGGCAGAGG 422

Matrix = 20p53g.matrix
Kimura (with divCpGMod) = 11.94
Transitions / transversions = 2.12 (34/16)
Gap_init rate = 0.12 (54 / 439), avg. gap size = 1.59 (86 / 54)

 1591   7.08 1.26 11.26  UnnamedSequence   1206  1683   1524 C SVA_F    Retroposon/SVA    421    855    520    
1591 7.08 1.26 11.26 UnnamedSequence 1206 1683 (1524) C SVA_F#Retroposon/SVA (520) 855 421 m_b1s502i7

  UnnamedSequen       1206 CCTCCCGGACGGGGCGGCTGGCCGGGCAGAGGGGCTCCTCACTTCCCAGT 1255
                                            -                                
C SVA_F#Retropo        855 CCTCCCGGACGGGGCGG-TGGCCGGGCAGAGGGGCTCCTCACTTCCCAGT 807

  UnnamedSequen       1256 AGGGGCGGCCGAGCAGAGGCGCCCCTCACCTCCCGGACGGGGCGGCTGGC 1305
                                      i                                      
C SVA_F#Retropo        806 AGGGGCGGCCGGGCAGAGGCGCCCCTCACCTCCCGGACGGGGCGGCTGGC 757

  UnnamedSequen       1306 CGGGCAGGGGGGCCGACCCCCCCCCCACCTCCCTCCCGGACAGGGCGGCT 1355
                                -       i  ---           -          i        
C SVA_F#Retropo        756 CGGGC-GGGGGGCTGA---CCCCCCCACCT-CCTCCCGGACGGGGCGGCT 712

  UnnamedSequen       1356 GGCCGGGCGGGGGGCCGACCCCCCCACCTCCCTCCCGGACGGGGCGGCTG 1405
                                          i          ----                    
C SVA_F#Retropo        711 GGCCGGGCGGGGGGCTGACCCCCCCA----CCTCCCGGACGGGGCGGCTG 666

  UnnamedSequen       1406 GCCGGGCGGGGGGCCGACCCCCCCACCTCCCTCCCGGACAGGGCGGCTGG 1455
                                    i  --  -- i  i  ---- ?        i    ----  
C SVA_F#Retropo        665 GCCGGGCGGAGG--CG--CTCCTCA----CNTCCCGGACGGGGC----GG 628

  UnnamedSequen       1456 CCGGGCAGAGGGGCTCCTCACTTCCCAGT--AGGGGCGGCCGGGCAGAGG 1503
                                 i    v         ?    i v--i?            i   i
C SVA_F#Retropo        627 CCGGGCGGAGGCGCTCCTCACNTCCCGGACGGNGGGCGGCCGGGCGGAGA 578

  UnnamedSequen       1504 CGCCCCTCACCTCCCGGACAGGGCGGCTGGCTGGGCGGGGGGGCTGACCC 1553
                              i               i       -   i      i  v   -----
C SVA_F#Retropo        577 CGCTCCTCACCTCCCGGACGGGGCGGC-GGCCGGGCGGAGGCGCT----- 534

  UnnamedSequen       1554 CCCCCAACCTCCCTCCCAGACGGGGCGGCTGGCCGGGC-GGGGGGCTGAC 1602
                           -  i  -----               ----        - i      ---
C SVA_F#Retropo        533 -CCTCA-----CCTCCCAGACGGGGC----GGCCGGGCAGAGGGGCT--- 497

  UnnamedSequen       1603 CCCCCCACCTCCCTCCCGGACGGGGCGGCTGGCCGGGCAGAGGGGCTCCT 1652
                           --  i  ---- ?    i    iv -   -             v      
C SVA_F#Retropo        496 --CCTCA----CNTCCCAGACGATG-GGC-GGCCGGGCAGAGGCGCTCCT 455

  UnnamedSequen       1653 CACTTCCCAGTAGG---GGCGGCCGGGCAGAGGC 1683
                              i      vv  ---                 
C SVA_F#Retropo        454 CACCTCCCAGACGGGGCGGCGGCCGGGCAGAGGC 421

Matrix = 20p53g.matrix
Kimura (with divCpGMod) = 5.83
Transitions / transversions = 3.29 (23/7)
Gap_init rate = 0.11 (52 / 477), avg. gap size = 1.06 (55 / 52)

 1014  10.06 2.03 15.14  UnnamedSequence   1282  1773   1434 C SVA_E    Retroposon/SVA    429    864    518    
1014 10.06 2.03 15.14 UnnamedSequence 1282 1773 (1434) C SVA_E#Retroposon/SVA (518) 864 429 m_b1s502i8

  UnnamedSequen       1282 CACCTCCCGGACGGGGCGGCTGGCCGGGCAGGGGGGCCGACCCCCCCCCC 1331
                                              -           i     i--------  i 
C SVA_E#Retropo        864 CACCTCCCGGACGGGGCGG-TGGCCGGGCAGAGGGGCT--------CCTC 824

  UnnamedSequen       1332 ACCTCCCTCCCGGACAGGGCGGCTGGCCGGGCGGGGGGCCGACCCCCCCA 1381
                             i    -----iii    --  -        i i  --  --    i  
C SVA_E#Retropo        823 ACTTCCC-----AGTAGGG--GC-GGCCGGGCAGAGG--CG--CCCCTCA 786

  UnnamedSequen       1382 CCTCCCTCCCGGACGGGGCGGCTGGCCGGGCGGGGGGCCGACCCCCCCAC 1431
                                 ----                            i           
C SVA_E#Retropo        785 CCTCCC----GGACGGGGCGGCTGGCCGGGCGGGGGGCTGACCCCCCCAC 740

  UnnamedSequen       1432 CTCCCTCCCGGACAGGGCGGCTGGCCGGGCAGAGGGGCT-----CCTCAC 1476
                               -        i                i -      -----  i   
C SVA_E#Retropo        739 CTCC-TCCCGGACGGGGCGGCTGGCCGGGCGG-GGGGCTGACCCCCCCAC 692

  UnnamedSequen       1477 TTCCCAGTAGGGGCGGC----CGGGCAGAGGCGCCCCTCACCTCCCGGAC 1522
                           i    i vv        ----     i       i               
C SVA_E#Retropo        691 CTCCCGGACGGGGCGGCTGGCCGGGCGGAGGCGCTCCTCACCTCCCGGAC 642

  UnnamedSequen       1523 AGGGCGGCTGGCTGGGCGGGGGGGCTGACCCCCCCCAACCTCCCTCCCAG 1572
                           i       ?  ----    i  v   ------     ----- ?    i 
C SVA_E#Retropo        641 GGGGCGGCNGG----GCGGAGGCGCT------CCCCA-----CNTCCCGG 607

  UnnamedSequen       1573 ACGGGGCGGCTGGCCGGGCGGGGG-GCTGACCCCCCCACCTCCCTCCCGG 1621
                              ?? -   -        i i  -   -----  i   i    ----i 
C SVA_E#Retropo        606 ACGNNG-GGC-GGCCGGGCAGAGGCGCT-----CCTCACTTCCC----AG 568

  UnnamedSequen       1622 ACGGGGCGGCTGGCCGGGCAGAGGGGCTCCTCACTTCCCAGTAGGGGCGG 1671
                                     -             v         i    i vv       
C SVA_E#Retropo        567 ACGGGGCGGC-GGCCGGGCAGAGGCGCTCCTCACCTCCCGGACGGGGCGG 519

  UnnamedSequen       1672 CCGGGCAGAGGCGCCCCTCACCTCCCGGACGGGGCGGCTGGCCGGGCAGG 1721
                                      v  i      ?         iv -   -          i
C SVA_E#Retropo        518 CCGGGCAGAGGGGCTCCTCACNTCCCGGACGATG-GGC-GGCCGGGCAGA 471

  UnnamedSequen       1722 GGGGCCGACCCCCCCACCTCCCTCCCGGACGGGGCGGCTGGCCGGGCAGA 1771
                             v ----- i  i  ----                  -           
C SVA_E#Retropo        470 GGCG-----CTCCTCA----CCTCCCGGACGGGGCGGC-GGCCGGGCAGA 431

  UnnamedSequen       1772 GG 1773
                             
C SVA_E#Retropo        430 GG 429

Matrix = 20p53g.matrix
Kimura (with divCpGMod) = 9.11
Transitions / transversions = 3.67 (33/9)
Gap_init rate = 0.14 (69 / 491), avg. gap size = 1.10 (76 / 69)

 1823   6.97 0.21 7.60  UnnamedSequence   1435  1900   1307 C SVA_F    Retroposon/SVA    422    855    520    
1823 6.97 0.21 7.60 UnnamedSequence 1435 1900 (1307) C SVA_F#Retroposon/SVA (520) 855 422 m_b1s502i9

  UnnamedSequen       1435 CCTCCCGGACAGGGCGGCTGGCCGGGCAGAGGGGCTCCTCACTTCCCAGT 1484
                                     i      -                                
C SVA_F#Retropo        855 CCTCCCGGACGGGGCGG-TGGCCGGGCAGAGGGGCTCCTCACTTCCCAGT 807

  UnnamedSequen       1485 AGGGGCGGCCGGGCAGAGGCGCCCCTCACCTCCCGGACAGGGCGGCTGGC 1534
                                                                 i           
C SVA_F#Retropo        806 AGGGGCGGCCGGGCAGAGGCGCCCCTCACCTCCCGGACGGGGCGGCTGGC 757

  UnnamedSequen       1535 TGGGCGGGGGGGCTGACCCCCCCCAACCTCCCTCCCAGACGGGGCGGCTG 1584
                           i    -                 --    -      i             
C SVA_F#Retropo        756 CGGGC-GGGGGGCTGACCCCCCC--ACCT-CCTCCCGGACGGGGCGGCTG 711

  UnnamedSequen       1585 GCCGGGCGGGGGGCTGACCCCCCCACCTCCCTCCCGGACGGGGCGGCTGG 1634
                                                    ----                     
C SVA_F#Retropo        710 GCCGGGCGGGGGGCTGACCCCCCCA----CCTCCCGGACGGGGCGGCTGG 665

  UnnamedSequen       1635 CCGGGCAGAGGGGCTCCTCACTTCCCAGTAGGGGCGGCCGGGCAGAGGCG 1684
                                 i    v         ?    i vv             i      
C SVA_F#Retropo        664 CCGGGCGGAGGCGCTCCTCACNTCCCGGACGGGGCGGCCGGGCGGAGGCG 615

  UnnamedSequen       1685 CCCCTCACCTCCCGGACGGGGCGGCTGGCCGGGCAGGGGGGCCGACCCCC 1734
                            i      ?          ? -   -        ---  i v  -- i  
C SVA_F#Retropo        614 CTCCTCACNTCCCGGACGGNG-GGC-GGCCGGGC---GGAGACG--CTCC 572

  UnnamedSequen       1735 CCACCTCCCTCCCGGACGGGGCGGCTGGCCGGGCAGAGGGGCTCCTCACT 1784
                           i  ----                  -        i    v         i
C SVA_F#Retropo        571 TCA----CCTCCCGGACGGGGCGGC-GGCCGGGCGGAGGCGCTCCTCACC 527

  UnnamedSequen       1785 TCCCAGTAGGGGCGGCCGGGCAGAGGCGCCCCTCACCTCCCAGACGGGGC 1834
                                 vv                  v  i      ?         iv -
C SVA_F#Retropo        526 TCCCAGACGGGGCGGCCGGGCAGAGGGGCTCCTCACNTCCCAGACGATG- 478

  UnnamedSequen       1835 GGCTGGCCGGGCGGAGG-GCTGACCCCCCCACCTCCCTCCCCGACAGGGC 1883
                              -        i    -   -----  i  ----      v   i    
C SVA_F#Retropo        477 GGC-GGCCGGGCAGAGGCGCT-----CCTCA----CCTCCCAGACGGGGC 438

  UnnamedSequen       1884 GGCTGGCCGGGCGGGGG 1900
                              -        i i  
C SVA_F#Retropo        437 GGC-GGCCGGGCAGAGG 422

Matrix = 20p53g.matrix
Kimura (with divCpGMod) = 5.30
Transitions / transversions = 2.00 (20/10)
Gap_init rate = 0.07 (34 / 465), avg. gap size = 1.00 (34 / 34)

  867  11.76 4.82 14.42  UnnamedSequence   1511  1987   1220 C SVA_E    Retroposon/SVA    428    864    518    
867 11.76 4.82 14.42 UnnamedSequence 1511 1987 (1220) C SVA_E#Retroposon/SVA (518) 864 428 m_b1s502i10

  UnnamedSequen       1511 CACCTCCCGGACAGGGCGGCTGGCTGGGCGGGGGGGCTGACCCCCCCCAA 1560
                                       i      -    i    i i      ------  i  -
C SVA_E#Retropo        864 CACCTCCCGGACGGGGCGG-TGGCCGGGCAGAGGGGCT------CCTCA- 823

  UnnamedSequen       1561 CCTCCCTCCCAGACGGGGCGGCTGGCCGGGCGGGGGGCTGACCCCCCCAC 1610
                           ---- i      vv        ----     i i  --i --    i   
C SVA_E#Retropo        822 ----CTTCCCAGTAGGGGCGGC----CGGGCAGAGG--CG--CCCCTCAC 785

  UnnamedSequen       1611 CTCCCTCCCGGACGGGGCGGCTGGCCGGGCAGAGGGGCT-----CCTCAC 1655
                                ----                     i -      -----  i   
C SVA_E#Retropo        784 CTCCC----GGACGGGGCGGCTGGCCGGGCGG-GGGGCTGACCCCCCCAC 740

  UnnamedSequen       1656 TTCC---CAGTAGGGGCGGC----CGGGCAGAGG--CG--CCCCTCACCT 1694
                           i   --- i vv        ----     i i  --i --    i     
C SVA_E#Retropo        739 CTCCTCCCGGACGGGGCGGCTGGCCGGGCGGGGGGCTGACCCCCCCACCT 690

  UnnamedSequen       1695 CCCGGACGGGGCGGCTGGCCGGGCAGGGGGGCCGACCCCCCCACCTCCCT 1744
                                                   i i  v  i-----  i        -
C SVA_E#Retropo        689 CCCGGACGGGGCGGCTGGCCGGGCGGAGGCGCT-----CCTCACCTCCC- 646

  UnnamedSequen       1745 CCCGGACGGGGCGGCTGGCCGGGCAGAGGGGCTCCTCACTTCCCAGT--A 1792
                           ---            ?  ----  i    v     i   ?    i v--?
C SVA_E#Retropo        645 ---GGACGGGGCGGCNGG----GCGGAGGCGCTCCCCACNTCCCGGACGN 603

  UnnamedSequen       1793 GGGGCGGCCGGGCAGAGGCGCCCCTCACCTCCCAGACGGGGCGGCTGGCC 1842
                           ?                    i      i                -    
C SVA_E#Retropo        602 NGGGCGGCCGGGCAGAGGCGCTCCTCACTTCCCAGACGGGGCGGC-GGCC 554

  UnnamedSequen       1843 GGGCGGAGG-GCTGACCCCCCCACCTCCCTCCCCGACAGGGCGGCTGGCC 1891
                               i    -   -----  i  ----      v   i    ----    
C SVA_E#Retropo        553 GGGCAGAGGCGCT-----CCTCA----CCTCCCGGACGGGGC----GGCC 517

  UnnamedSequen       1892 GGGC-GGGGGGCTGACCCCCCCACCTCCCTCCCGGACGGGGCGGCTGGCC 1940
                               - i      -----  i  ---- ?         iv -   -    
C SVA_E#Retropo        516 GGGCAGAGGGGCT-----CCTCA----CNTCCCGGACGATG-GGC-GGCC 478

  UnnamedSequen       1941 GGGCAGAGGGGCTCCTCACTTCCCAGTAGG---GGCGGCTGGGCAGAGGC 1987
                                    v         i    i vv  ---      i          
C SVA_E#Retropo        477 GGGCAGAGGCGCTCCTCACCTCCCGGACGGGGCGGCGGCCGGGCAGAGGC 428

Matrix = 20p53g.matrix
Kimura (with divCpGMod) = 9.79
Transitions / transversions = 3.00 (36/12)
Gap_init rate = 0.15 (72 / 476), avg. gap size = 1.19 (86 / 72)

 1492   7.87 2.59 9.45  UnnamedSequence   1614  2076   1131 C SVA_F    Retroposon/SVA    422    855    520    
1492 7.87 2.59 9.45 UnnamedSequence 1614 2076 (1131) C SVA_F#Retroposon/SVA (520) 855 422 m_b1s502i11

  UnnamedSequen       1614 CCTCCCGGACGGGGCGGCTGGCCGGGCAGAGGGGCTCCTCACTTCCCAGT 1663
                                            -                                
C SVA_F#Retropo        855 CCTCCCGGACGGGGCGG-TGGCCGGGCAGAGGGGCTCCTCACTTCCCAGT 807

  UnnamedSequen       1664 AGGGGCGGCCGGGCAGAGGCGCCCCTCACCTCCCGGACGGGGCGGCTGGC 1713
                                                                             
C SVA_F#Retropo        806 AGGGGCGGCCGGGCAGAGGCGCCCCTCACCTCCCGGACGGGGCGGCTGGC 757

  UnnamedSequen       1714 CGGGCAGGGGGGCCGACCCCCCCACCTCCCTCCCGGACGGGGCGGCTGGC 1763
                                -       i             -                      
C SVA_F#Retropo        756 CGGGC-GGGGGGCTGACCCCCCCACCT-CCTCCCGGACGGGGCGGCTGGC 709

  UnnamedSequen       1764 CGGGCAGAGGGGCT-----CCTCACTTCCCAGTAGGGGC----GGCCGGG 1804
                                - i      -----  i   i    i vv     ----       
C SVA_F#Retropo        708 CGGGC-GGGGGGCTGACCCCCCCACCTCCCGGACGGGGCGGCTGGCCGGG 660

  UnnamedSequen       1805 CAGAGGCGCCCCTCACCTCCCAGACGGGGCGGCTGGCCGGGCGGAGG-GC 1853
                            i       i      ?    i        ----             -  
C SVA_F#Retropo        659 CGGAGGCGCTCCTCACNTCCCGGACGGGGC----GGCCGGGCGGAGGCGC 614

  UnnamedSequen       1854 TGACCCCCCCACCTCCCTCCCCGACAGGGCGGCTGGCCGGGCGGGGGGCT 1903
                            -----  i  ---- ?    v   --  ?  vi          i-----
C SVA_F#Retropo        613 T-----CCTCA----CNTCCCGGAC--GGNGGGCGGCCGGGCGGA----- 580

  UnnamedSequen       1904 GAC-CCCCCCACCTCCCTCCCGGACGGGGCGGCTGGCCGGGCAGAGGGGC 1952
                              - i  i  ----                  -        i    v  
C SVA_F#Retropo        579 GACGCTCCTCA----CCTCCCGGACGGGGCGGC-GGCCGGGCGGAGGCGC 535

  UnnamedSequen       1953 TCCTCACTTCCCAGTAGGGGCGGCTGGGCAGAGGCGCCCCTCACCTCCCA 2002
                                  i      vv        i         v  i      ?     
C SVA_F#Retropo        534 TCCTCACCTCCCAGACGGGGCGGCCGGGCAGAGGGGCTCCTCACNTCCCA 485

  UnnamedSequen       2003 GACGGGGCGGCTGGCCGGGCGGAGG-GCTGACCCCCCCACCTCCCTCCCG 2051
                               iv -   -        i    -   -----  i  ----      i
C SVA_F#Retropo        484 GACGATG-GGC-GGCCGGGCAGAGGCGCT-----CCTCA----CCTCCCA 446

  UnnamedSequen       2052 GACGGGGCGGCTGGCCAGGCGGGGG 2076
                                      -    i   i i  
C SVA_F#Retropo        445 GACGGGGCGGC-GGCCGGGCAGAGG 422

Matrix = 20p53g.matrix
Kimura (with divCpGMod) = 6.72
Transitions / transversions = 2.67 (24/9)
Gap_init rate = 0.10 (46 / 462), avg. gap size = 1.15 (53 / 46)

  988  11.36 3.09 14.91  UnnamedSequence   1690  2175   1032 C SVA_E    Retroposon/SVA    429    864    518    
988 11.36 3.09 14.91 UnnamedSequence 1690 2175 (1032) C SVA_E#Retroposon/SVA (518) 864 429 m_b1s502i12

  UnnamedSequen       1690 CACCTCCCGGACGGGGCGGCTGGCCGGGCAGGGGGGCCGACCCCCCCACC 1739
                                              -           i    ----- i  i  --
C SVA_E#Retropo        864 CACCTCCCGGACGGGGCGG-TGGCCGGGCAGAGGGG-----CTCCTCA-- 823

  UnnamedSequen       1740 TCCCTCCCGGACGGGGCGGCTGGCCGGGCAGAGGGGCTCCTCACTTCCCA 1789
                           -- i    i vv     ----             v  i      i    i
C SVA_E#Retropo        822 --CTTCCCAGTAGGGGC----GGCCGGGCAGAGGCGCCCCTCACCTCCCG 779

  UnnamedSequen       1790 GTAGGGGC----GGCCGGGCAGAGG--CG--CCCCTCA---CCTCCCAGA 1828
                            vv     ----        i i  --i --    i  ---      i  
C SVA_E#Retropo        778 GACGGGGCGGCTGGCCGGGCGGGGGGCTGACCCCCCCACCTCCTCCCGGA 729

  UnnamedSequen       1829 CGGGGCGGCTGGCCGGGCGGAGGGCTGACCCCCCCACCTCCCTCCCCGAC 1878
                                               i               ----      v   
C SVA_E#Retropo        728 CGGGGCGGCTGGCCGGGCGGGGGGCTGACCCCCCCA----CCTCCCGGAC 683

  UnnamedSequen       1879 AGGGCGGCTGGCCGGGCGGGGG-GCTGACCCCCCCACCTCCCTCCCGGAC 1927
                           i                  i  -   -----  i  ----          
C SVA_E#Retropo        682 GGGGCGGCTGGCCGGGCGGAGGCGCT-----CCTCA----CCTCCCGGAC 642

  UnnamedSequen       1928 GGGGCGGCTGGCCGGGCAGAGGGGCTCCTCACTTCCCAGT--AGGGGCGG 1975
                                ----   ?    i    v     i   ?    i v--??      
C SVA_E#Retropo        641 GGGGC----GGCNGGGCGGAGGCGCTCCCCACNTCCCGGACGNNGGGCGG 596

  UnnamedSequen       1976 CTGGGCAGAGGCGCCCCTCACCTCCCAGACGGGGCGGCTGGCCGGGCGGA 2025
                            i            i      i                -        i  
C SVA_E#Retropo        595 CCGGGCAGAGGCGCTCCTCACTTCCCAGACGGGGCGGC-GGCCGGGCAGA 547

  UnnamedSequen       2026 GG-GCTGACCCCCCCACCTCCCTCCCGGACGGGGCGGCTGGCCAGGCGGG 2074
                             -   -----  i  ----                  i  v  --- i 
C SVA_E#Retropo        546 GGCGCT-----CCTCA----CCTCCCGGACGGGGCGGCCGGGCA---GAG 509

  UnnamedSequen       2075 GGGCTGACCCCCCTACCTCCCTACCGGACGGGGCGGCTGGCCGGGTGGGG 2124
                                -----  ii  ?  ----       iv -   -       ii i 
C SVA_E#Retropo        508 GGGCT-----CCTCACNTC----CCGGACGATG-GGC-GGCCGGGCAGAG 470

  UnnamedSequen       2125 GGGCTGACCCCCCCATCTCCCTCCCGGACGGGGTGGCTGGCCGGGCTGAG 2174
                            v   -----  i  ----              i   -        v   
C SVA_E#Retropo        469 GCGCT-----CCTCA----CCTCCCGGACGGGGCGGC-GGCCGGGCAGAG 430

  UnnamedSequen       2175 G 2175
                            
C SVA_E#Retropo        429 G 429

Matrix = 20p53g.matrix
Kimura (with divCpGMod) = 10.09
Transitions / transversions = 2.92 (35/12)
Gap_init rate = 0.15 (72 / 485), avg. gap size = 1.11 (80 / 72)

 1754   7.74 1.06 9.66  UnnamedSequence   1742  2213    994 C SVA_F    Retroposon/SVA    421    855    520    
1754 7.74 1.06 9.66 UnnamedSequence 1742 2213 (994) C SVA_F#Retroposon/SVA (520) 855 421 m_b1s502i13

  UnnamedSequen       1742 CCTCCCGGACGGGGCGGCTGGCCGGGCAGAGGGGCTCCTCACTTCCCAGT 1791
                                            -                                
C SVA_F#Retropo        855 CCTCCCGGACGGGGCGG-TGGCCGGGCAGAGGGGCTCCTCACTTCCCAGT 807

  UnnamedSequen       1792 AGGGGCGGCCGGGCAGAGGCGCCCCTCACCTCCCAGACGGGGCGGCTGGC 1841
                                                             i               
C SVA_F#Retropo        806 AGGGGCGGCCGGGCAGAGGCGCCCCTCACCTCCCGGACGGGGCGGCTGGC 757

  UnnamedSequen       1842 CGGGCGGAGGGCTGACCCCCCCACCTCCCTCCCCGACAGGGCGGCTGGCC 1891
                                  i                  -      v   i            
C SVA_F#Retropo        756 CGGGCGGGGGGCTGACCCCCCCACCT-CCTCCCGGACGGGGCGGCTGGCC 708

  UnnamedSequen       1892 GGGCGGGGGGCTGACCCCCCCACCTCCCTCCCGGACGGGGCGGCTGGCCG 1941
                                                 ----                        
C SVA_F#Retropo        707 GGGCGGGGGGCTGACCCCCCCA----CCTCCCGGACGGGGCGGCTGGCCG 662

  UnnamedSequen       1942 GGCAGAGGGGCTCCTCACTTCCCAGTAGGGGCGGCTGGGCAGAGGCGCCC 1991
                              i    v         ?    i vv        i    i       i 
C SVA_F#Retropo        661 GGCGGAGGCGCTCCTCACNTCCCGGACGGGGCGGCCGGGCGGAGGCGCTC 612

  UnnamedSequen       1992 CTCACCTCCCAGACGGGGCGGCTGGCCGGGCGGAGGGCTGAC-CCCCCCA 2040
                                ?    i     ? -   -           -----   - i  i  
C SVA_F#Retropo        611 CTCACNTCCCGGACGGNG-GGC-GGCCGGGCGGA-----GACGCTCCTCA 569

  UnnamedSequen       2041 CCTCCCTCCCGGACGGGGCGGCTGGCCAGGCGGGGG-GCTGACCCCCCTA 2089
                           ----                  -    i     i  -   -----  ii 
C SVA_F#Retropo        568 ----CCTCCCGGACGGGGCGGC-GGCCGGGCGGAGGCGCT-----CCTCA 529

  UnnamedSequen       2090 CCTCCCTACCGGACGGGGCGGCTGGCCGGGTGGGGGGGCTGACCCCCCCA 2139
                               ----  i        ----       ii i      -----  i  
C SVA_F#Retropo        528 CCTC----CCAGACGGGGC----GGCCGGGCAGAGGGGCT-----CCTCA 492

  UnnamedSequen       2140 TCTCCCTCCCGGACGGGGTGGCTGGCCGGGCTGAGGGGCTCCTCACTTCC 2189
                           ---- ?    i   -- i   vi        v    v         i   
C SVA_F#Retropo        491 ----CNTCCCAGAC--GATGGGCGGCCGGGCAGAGGCGCTCCTCACCTCC 448

  UnnamedSequen       2190 CAGTAGG---GGCGGCCGGGCAGAGGC 2213
                              vv  ---                 
C SVA_F#Retropo        447 CAGACGGGGCGGCGGCCGGGCAGAGGC 421

Matrix = 20p53g.matrix
Kimura (with divCpGMod) = 6.11
Transitions / transversions = 2.67 (24/9)
Gap_init rate = 0.10 (45 / 471), avg. gap size = 1.04 (47 / 45)

  680  12.95 3.92 15.60  UnnamedSequence   1818  2302    905 C SVA_E    Retroposon/SVA    429    864    518    
680 12.95 3.92 15.60 UnnamedSequence 1818 2302 (905) C SVA_E#Retroposon/SVA (518) 864 429 m_b1s502i14

  UnnamedSequen       1818 CACCTCCCAGACGGGGCGGCTGGCCGGGCGGAGGGCTGACCCCCCCACCT 1867
                                   i          -         i     v--------- v   
C SVA_E#Retropo        864 CACCTCCCGGACGGGGCGG-TGGCCGGGCAGAGGGG---------CTCCT 825

  UnnamedSequen       1868 CCCTCCCCGACAGGGCGGCTGGCCGGGCGGGGGGCTGACCCCCCCACCTC 1917
                            v  i   iii    --  -        i i  --i --    i      
C SVA_E#Retropo        824 CACTTCCCAGTAGGG--GC-GGCCGGGCAGAGG--CG--CCCCTCACCTC 782

  UnnamedSequen       1918 CCTCCCGGACGGGGCGGCTGGCCGGGCAGAGGGGCT-----CCTCACTTC 1962
                             ----                     i -      -----  i   i  
C SVA_E#Retropo        781 CC----GGACGGGGCGGCTGGCCGGGCGG-GGGGCTGACCCCCCCACCTC 737

  UnnamedSequen       1963 C---CAGTAGGGGCGGCTGG----GCAGAGG--CG--CCCCTCACCTCCC 2001
                            --- i vv           ----  i i  --i --    i        
C SVA_E#Retropo        736 CTCCCGGACGGGGCGGCTGGCCGGGCGGGGGGCTGACCCCCCCACCTCCC 687

  UnnamedSequen       2002 AGACGGGGCGGCTGGCCGGGCGGAGG-GCTGACCCCCCCACCTCCCTCCC 2050
                           i                         -   -----  i        ----
C SVA_E#Retropo        686 GGACGGGGCGGCTGGCCGGGCGGAGGCGCT-----CCTCACCTCCC---- 646

  UnnamedSequen       2051 GGACGGGGCGGCTGGCCAGGCGGGGG-GCTGACCCCCCTACCTCCCTACC 2099
                                       ?  ----    i  -   -----   i  ?    ----
C SVA_E#Retropo        645 GGACGGGGCGGCNGG----GCGGAGGCGCT-----CCCCACNTCCC---- 609

  UnnamedSequen       2100 GGACGGGGCGGCTGGCCGGGTGGGGGGGCTGACCCCCCCATCTCCCTCCC 2149
                                ?? -   -       ii i  v   -----  i  ---- i    
C SVA_E#Retropo        608 GGACGNNG-GGC-GGCCGGGCAGAGGCGCT-----CCTCA----CTTCCC 570

  UnnamedSequen       2150 GGACGGGGTGGCTGGCCGGGCTGAGGGGCTCCTCACTTCCCAGTAGGGGC 2199
                           i       i   -        v    v         i    i vv     
C SVA_E#Retropo        569 AGACGGGGCGGC-GGCCGGGCAGAGGCGCTCCTCACCTCCCGGACGGGGC 521

  UnnamedSequen       2200 GGCCGGGCAGAGGCGCCCCTCACCTCCCGGACGGGGCGGCTGGCCGGGCG 2249
                                        v  i      ?         iv -   -        i
C SVA_E#Retropo        520 GGCCGGGCAGAGGGGCTCCTCACNTCCCGGACGATG-GGC-GGCCGGGCA 473

  UnnamedSequen       2250 GGGG-GCTGACCCCCCCACCTCCCTCCCGGATGGCACGGCTGGCCGGGCG 2298
                            i  -   -----  i  ----         i  vi    -        i
C SVA_E#Retropo        472 GAGGCGCT-----CCTCA----CCTCCCGGACGGGGCGGC-GGCCGGGCA 433

  UnnamedSequen       2299 GGGG 2302
                            i  
C SVA_E#Retropo        432 GAGG 429

Matrix = 20p53g.matrix
Kimura (with divCpGMod) = 11.94
Transitions / transversions = 3.08 (40/13)
Gap_init rate = 0.16 (76 / 484), avg. gap size = 1.14 (87 / 76)

 1543   9.16 0.62 12.41  UnnamedSequence   1918  2403    804 C SVA_F    Retroposon/SVA    421    855    520    
1543 9.16 0.62 12.41 UnnamedSequence 1918 2403 (804) C SVA_F#Retroposon/SVA (520) 855 421 m_b1s502i15

  UnnamedSequen       1918 CCTCCCGGACGGGGCGGCTGGCCGGGCAGAGGGGCTCCTCACTTCCCAGT 1967
                                            -                                
C SVA_F#Retropo        855 CCTCCCGGACGGGGCGG-TGGCCGGGCAGAGGGGCTCCTCACTTCCCAGT 807

  UnnamedSequen       1968 AGGGGCGGCTGGGCAGAGGCGCCCCTCACCTCCCAGACGGGGCGGCTGGC 2017
                                    i                        i               
C SVA_F#Retropo        806 AGGGGCGGCCGGGCAGAGGCGCCCCTCACCTCCCGGACGGGGCGGCTGGC 757

  UnnamedSequen       2018 CGGGCGGAGGGCTGACCCCCCCACCTCCCTCCCGGACGGGGCGGCTGGCC 2067
                                  i                  -                       
C SVA_F#Retropo        756 CGGGCGGGGGGCTGACCCCCCCACCT-CCTCCCGGACGGGGCGGCTGGCC 708

  UnnamedSequen       2068 AGGCGGGGGGCTGACCCCCCTACCTCCCTACCGGACGGGGCGGCTGGCCG 2117
                           i                   i     ----                    
C SVA_F#Retropo        707 GGGCGGGGGGCTGACCCCCCCACCTC----CCGGACGGGGCGGCTGGCCG 662

  UnnamedSequen       2118 GGTGGGGGGGCTGACCCCCCCATCTCCCTCCCGGACGGGGTGGCTGGCCG 2167
                             i  i  v   -----  i  ---- ?            ----i     
C SVA_F#Retropo        661 GGCGGAGGCGCT-----CCTCA----CNTCCCGGACGGGG----CGGCCG 625

  UnnamedSequen       2168 GGCTGAGGGGCTCCTCACTTCCCAGT--AGGGGCGGCCGGGCAGAGGCGC 2215
                              v    v         ?    i v--i?            i   i   
C SVA_F#Retropo        624 GGCGGAGGCGCTCCTCACNTCCCGGACGGNGGGCGGCCGGGCGGAGACGC 575

  UnnamedSequen       2216 CCCTCACCTCCCGGACGGGGCGGCTGGCCGGGCGGGGG-GCTGACCCCCC 2264
                           i                       -          i  -   -----  i
C SVA_F#Retropo        574 TCCTCACCTCCCGGACGGGGCGGC-GGCCGGGCGGAGGCGCT-----CCT 531

  UnnamedSequen       2265 CACCTCCCTCCCGGATGGCACGGCTGGCCGGGCGGGGGGGCTGACCCCCC 2314
                             ----      i  i  ---   -        i i      -----  i
C SVA_F#Retropo        530 CA----CCTCCCAGACGG---GGC-GGCCGGGCAGAGGGGCT-----CCT 494

  UnnamedSequen       2315 CACCTCCCTCCCGGATGGGGCGGCTGGCCGGGCGGGGGCTGACCCCCCCC 2364
                              ?    vvv -      -----i        i i  ------ v i  
C SVA_F#Retropo        493 CACNTCCCAGAC-GATGGG-----CGGCCGGGCAGAGG------CGCTCC 456

  UnnamedSequen       2365 CCACCTCCCTCCTGGACGGGGTGGCTGCCGGGCGGAGAC 2403
                           i        ----i       i   v       i   i 
C SVA_F#Retropo        455 TCACCTCCC----AGACGGGGCGGCGGCCGGGCAGAGGC 421

Matrix = 20p53g.matrix
Kimura (with divCpGMod) = 7.42
Transitions / transversions = 3.33 (30/9)
Gap_init rate = 0.12 (56 / 485), avg. gap size = 1.02 (57 / 56)

 1057  13.86 2.04 14.45  UnnamedSequence   1994  2482    725 C SVA_A    Retroposon/SVA    437    872    515    
1057 13.86 2.04 14.45 UnnamedSequence 1994 2482 (725) C SVA_A#Retroposon/SVA (515) 872 437 m_b1s502i16

  UnnamedSequen       1994 CACCTCCCAGACGGGGCGGCTGGCCGGGCGGAGGGCTGACCCCCCCACCT 2043
                                   i           -        i     v---- i  i   i 
C SVA_A#Retropo        872 CACCTCCCGGACGGGGCGGC-GGCCGGGCAGAGGGG----CTCCTCACTT 828

  UnnamedSequen       2044 CCCTCCCGGACGGGGCGGCTGGCCAGGCGGGGGGCTGACCCCCCTACCTC 2093
                              ----i ??        i  v   iv v --------    ii     
C SVA_A#Retropo        827 CCC----AGNNGGGGCGGCCGGGCAGAGGCG--------CCCCTCACCTC 790

  UnnamedSequen       2094 CCTACCGGACGGGGCGGCTGGCCGGGTGGGGGGGCTGACCCCCCCATCTC 2143
                             ----                    i      -            i   
C SVA_A#Retropo        789 CC----GGACGGGGCGGCTGGCCGGGCGGGGGG-CTGACCCCCCCACCTC 745

  UnnamedSequen       2144 CCTCCCGGACGGGGTGGCTGGCCGGGCTGAGGGGCT-----CCTCACTTC 2188
                            -            i            - i      -----  i   i  
C SVA_A#Retropo        744 C-TCCCGGACGGGGCGGCTGGCCGGGC-GGGGGGCTGACCCCCCCACCTC 697

  UnnamedSequen       2189 CCAGTAGGGGCGGC----CGGGCAGAGGCGCCCCTCACCTCCCGGACGGG 2234
                             i vv        ----     i       i      ?    i      
C SVA_A#Retropo        696 CCGGACGGGGCGGCTGGCCGGGCGGAGGCGCTCCTCACNTCCCAGACGGG 647

  UnnamedSequen       2235 GCGGCTGGCCGGGCGGGGG-GCTGACCCCCCCACCTCCCTCCCGGATGGC 2283
                                ----       i  -   -----  i   ?    ----i  i --
C SVA_A#Retropo        646 GCGGC----CGGGCGGAGGCGCT-----CCTCACNTCCC----AGACG-- 612

  UnnamedSequen       2284 ACGGCTGGCCGGGCGGGGGGGCTGACCCCCCCACCTCCCTCCCGGATGGG 2333
                            ?  vi        i i  v   -----  i   i    ----i  i   
C SVA_A#Retropo        611 ANGGGCGGCCGGGCAGAGGCGCT-----CCTCACTTCCC----AGACGGG 571

  UnnamedSequen       2334 GCGGCTGGCCGGGCGGGGGCTGACCCCCCCCCCACCTCCCTCCTGGACGG 2383
                                -        i i   v------ i  i   i    ----i     
C SVA_A#Retropo        570 GCGGC-GGCCGGGCAGAGGCG------CTCCTCACTTCCC----AGACGG 532

  UnnamedSequen       2384 GGTGGCTGCCGGGCGGAGACGCTCCTCACTTCCCAGATGGGGTGGCTGCC 2433
                             i   ---     i   i          ?       i iv -   v   
C SVA_A#Retropo        531 GGCGGC---CGGGCAGAGGCGCTCCTCACNTCCCAGACGATG-GGCGGCC 486

  UnnamedSequen       2434 GGGCAGAGACGCTCCTCACTTCTCAGACGGGGCAGCTGCCGGGCGGAGG 2482
                                                 i         ii  v       i    
C SVA_A#Retropo        485 GGGCAGAGACGCTCCTCACTTCCCAGACGGGGTGGCGGCCGGGCAGAGG 437

Matrix = 20p53g.matrix
Kimura (with divCpGMod) = 12.66
Transitions / transversions = 3.83 (46/12)
Gap_init rate = 0.14 (66 / 488), avg. gap size = 1.11 (73 / 66)

 1094  14.46 9.32 8.06  UnnamedSequence   2094  2522    685 C SVA_F    Retroposon/SVA    422    855    520    
1094 14.46 9.32 8.06 UnnamedSequence 2094 2522 (685) C SVA_F#Retroposon/SVA (520) 855 422 m_b1s502i17

  UnnamedSequen       2094 CCTACCGGACGGGGCGGCTGGCCGGGTGGGGGGGCTGACCCCCCCATCTC 2143
                              v             -        ii i      -----  i  ----
C SVA_F#Retropo        855 CCTCCCGGACGGGGCGG-TGGCCGGGCAGAGGGGCT-----CCTCA---- 816

  UnnamedSequen       2144 CCTCCCGGACGGGGTGGCTGGCCGGGCTGAGGGGCTCCTCACTTCCCAGT 2193
                            i    i vv    i   ----     v    v  i      i    i v
C SVA_F#Retropo        815 CTTCCCAGTAGGGGCGGC----CGGGCAGAGGCGCCCCTCACCTCCCGGA 770

  UnnamedSequen       2194 AGGGGCGGC----CGGGCAGAGG--CG--CCCCTCACCTCC---CGGACG 2232
                           v        ----     i i  --i --    i       ---      
C SVA_F#Retropo        769 CGGGGCGGCTGGCCGGGCGGGGGGCTGACCCCCCCACCTCCTCCCGGACG 720

  UnnamedSequen       2233 GGGCGGCTGGCCGGGCGGGGGGCTGACCCCCCCACCTCCCTCCCGGATGG 2282
                                                                   ----   i  
C SVA_F#Retropo        719 GGGCGGCTGGCCGGGCGGGGGGCTGACCCCCCCACCTCCC----GGACGG 674

  UnnamedSequen       2283 CACGGCTGGCCGGGCGGGGGGGCTGACCCCCCCACCTCCCTCCCGGATGG 2332
                           vi               i  v   -----  i   ?    ----   i  
C SVA_F#Retropo        673 GGCGGCTGGCCGGGCGGAGGCGCT-----CCTCACNTCCC----GGACGG 633

  UnnamedSequen       2333 GGCGGCTGG--------------------CCGGGCGGGGGCTGACC---- 2358
                                 i  --------------------    i   ?  vi i  ----
C SVA_F#Retropo        632 GGCGGCCGGGCGGAGGCGCTCCTCACNTCCCGGACGGNGGGCGGCCGGGC 583

  UnnamedSequen       2359 -----CCCCCCCCACCTCCCTCCTGGACGGGGTGGCTGCCGGGCGGAGAC 2403
                           ----- v i  i        ----        i   v           i 
C SVA_F#Retropo        582 GGAGACGCTCCTCACCTCCC----GGACGGGGCGGCGGCCGGGCGGAGGC 537

  UnnamedSequen       2404 GCTCCTCACTTCCCAGATGGGGTGGCTGCCGGGCAGAGACGCTCCTCACT 2453
                                    i       i    i ---           iv         ?
C SVA_F#Retropo        536 GCTCCTCACCTCCCAGACGGGGCG---GCCGGGCAGAGGGGCTCCTCACN 490

  UnnamedSequen       2454 TCTCAGACGGGGCAGCTGCCGGGCGGAGGGGCTCCTCACTTCTCAGACGG 2503
                             i      iv -i  v       i    v         i  i       
C SVA_F#Retropo        489 TCCCAGACGATG-GGCGGCCGGGCAGAGGCGCTCCTCACCTCCCAGACGG 441

  UnnamedSequen       2504 GGTGGTTGCCAGGCAGAGG 2522
                             i  iv   i        
C SVA_F#Retropo        440 GGCGGCGGCCGGGCAGAGG 422

Matrix = 20p53g.matrix
Kimura (with divCpGMod) = 12.70
Transitions / transversions = 2.35 (40/17)
Gap_init rate = 0.10 (42 / 428), avg. gap size = 1.79 (75 / 42)

 2245   7.64 0.44 5.52  UnnamedSequence   2144  2600    607 C SVA_F    Retroposon/SVA    421    855    520    
2245 7.64 0.44 5.52 UnnamedSequence 2144 2600 (607) C SVA_F#Retroposon/SVA (520) 855 421 m_b1s502i18

  UnnamedSequen       2144 CCTCCCGGACGGGGTGGCTGGCCGGGCTGAGGGGCTCCTCACTTCCCAGT 2193
                                         i  -         v                      
C SVA_F#Retropo        855 CCTCCCGGACGGGGCGG-TGGCCGGGCAGAGGGGCTCCTCACTTCCCAGT 807

  UnnamedSequen       2194 AGGGGCGGCCGGGCAGAGGCGCCCCTCACCTCCCGGACGGGGCGGCTGGC 2243
                                                                             
C SVA_F#Retropo        806 AGGGGCGGCCGGGCAGAGGCGCCCCTCACCTCCCGGACGGGGCGGCTGGC 757

  UnnamedSequen       2244 CGGGCGGGGGGCTGACCCCCCCACCTCCCTCCCGGATGGCACGGCTGGCC 2293
                                                     -         i  vi         
C SVA_F#Retropo        756 CGGGCGGGGGGCTGACCCCCCCACCT-CCTCCCGGACGGGGCGGCTGGCC 708

  UnnamedSequen       2294 GGGCGGGGGGGCTGACCCCCCCACCTCCCTCCCGGATGGGGCGGCTGGCC 2343
                               -                  ----         i             
C SVA_F#Retropo        707 GGGC-GGGGGGCTGACCCCCCCA----CCTCCCGGACGGGGCGGCTGGCC 663

  UnnamedSequen       2344 GGGCGGGGGCTGACCCCCCCCCCACCTCCCTCCTGGACGGGGTGGCTGCC 2393
                                 i  ------ v i  i   ?    ----        i ---   
C SVA_F#Retropo        662 GGGCGGAGG------CGCTCCTCACNTCCC----GGACGGGGCG---GCC 626

  UnnamedSequen       2394 GGGCGGAGACGCTCCTCACTTCCCAGATGGGGTGGCTGCCGGGCAGAGAC 2443
                                   i          ?    i  i  ? -   v       i     
C SVA_F#Retropo        625 GGGCGGAGGCGCTCCTCACNTCCCGGACGGNG-GGCGGCCGGGCGGAGAC 577

  UnnamedSequen       2444 GCTCCTCACTTCTCAGACGGGGCAGCTGCCGGGCGGAGGGGCTCCTCACT 2493
                                    i  i i        i  v            v         i
C SVA_F#Retropo        576 GCTCCTCACCTCCCGGACGGGGCGGCGGCCGGGCGGAGGCGCTCCTCACC 527

  UnnamedSequen       2494 TCTCAGACGGGGTGGTTGCCAGGCAGAGGGTCTCCTCACTTCTCAGACG- 2542
                             i         i ---   i         v        ?  i      -
C SVA_F#Retropo        526 TCCCAGACGGGGCG---GCCGGGCAGAGGGGCTCCTCACNTCCCAGACGA 480

  UnnamedSequen       2543 -GGGCGGCCGAGCAGAGACGCTCCTCACCTCCCAGACGGGGTCTCGGCCG 2591
                           -         i      i                       ivv      
C SVA_F#Retropo        479 TGGGCGGCCGGGCAGAGGCGCTCCTCACCTCCCAGACGGGGCGGCGGCCG 430

  UnnamedSequen       2592 GGCAGAGGC 2600
                                    
C SVA_F#Retropo        429 GGCAGAGGC 421

Matrix = 20p53g.matrix
Kimura (with divCpGMod) = 5.38
Transitions / transversions = 2.67 (24/9)
Gap_init rate = 0.06 (26 / 456), avg. gap size = 1.00 (26 / 26)

 1706  12.73 4.04 6.67  UnnamedSequence   2271  2716    491 C SVA_A    Retroposon/SVA    436    870    517    
1706 12.73 4.04 6.67 UnnamedSequence 2271 2716 (491) C SVA_A#Retroposon/SVA (517) 870 436 m_b1s502i19

  UnnamedSequen       2271 CCTCCCGGATGGCACGGCTGGCCGGGCGGGGGGGCTGACCCCCCCACCTC 2320
                                    i  vi    -        i i      -----  i   i  
C SVA_A#Retropo        870 CCTCCCGGACGGGGCGGC-GGCCGGGCAGAGGGGCT-----CCTCACTTC 827

  UnnamedSequen       2321 CCTCCCGGATGGGGCGGCTGGCCGGGCGGGGGCTGACCCCCCCCCCACCT 2370
                             ----i ??        ----     i i   v------    i     
C SVA_A#Retropo        826 CC----AGNNGGGGCGGC----CGGGCAGAGGCG------CCCCTCACCT 791

  UnnamedSequen       2371 CCCTCCTGGACGGGGTGGCTG-CCGGGCGGAGACGCT-----CCTCACTT 2414
                              ----        i     -        i i-   -----  i   i 
C SVA_A#Retropo        790 CCC----GGACGGGGCGGCTGGCCGGGCGGGGG-GCTGACCCCCCCACCT 746

  UnnamedSequen       2415 CC---CAGATGGGGTGGCTG-CCGGGCAGAGACGCT-----CCTCACTTC 2455
                             --- i  i    i     -      i i i-   -----  i   i  
C SVA_A#Retropo        745 CCTCCCGGACGGGGCGGCTGGCCGGGCGGGGG-GCTGACCCCCCCACCTC 697

  UnnamedSequen       2456 TCAGACGGGGCAGCTG-CCGGGCGGAGGGGCTCCTCACTTCTCAGACGGG 2504
                           i i        i    -           v         ?  i        
C SVA_A#Retropo        696 CCGGACGGGGCGGCTGGCCGGGCGGAGGCGCTCCTCACNTCCCAGACGGG 647

  UnnamedSequen       2505 GTGGTTGCCAGGCAGAGGGTCTCCTCACTTCTCAGACG--GGGCGGCCGA 2552
                            i  ---  i   i    vv        ?  i      --         i
C SVA_A#Retropo        646 GCGG---CCGGGCGGAGGCGCTCCTCACNTCCCAGACGANGGGCGGCCGG 600

  UnnamedSequen       2553 GCAGAGACGCTCCTCACCTCCCAGACGGGGTCTCGGCCGGGCAGAGGCGC 2602
                                 i          i            ivv                 
C SVA_A#Retropo        599 GCAGAGGCGCTCCTCACTTCCCAGACGGGGCGGCGGCCGGGCAGAGGCGC 550

  UnnamedSequen       2603 TCCTCACATCCCAGATGGGGCGGCGGGGCAGAGGCGCTCCCCACATCTCA 2652
                                  v       i        v               i   ?  i  
C SVA_A#Retropo        549 TCCTCACTTCCCAGACGGGGCGGCCGGGCAGAGGCGCTCCTCACNTCCCA 500

  UnnamedSequen       2653 GACGATGGGCGGCCGGGCAGAGACGCTCCTCACTTCCTAGATGTGATGGC 2702
                                                                i   i v i    
C SVA_A#Retropo        499 GACGATGGGCGGCCGGGCAGAGACGCTCCTCACTTCCCAGACGGGGTGGC 450

  UnnamedSequen       2703 GGCTGGGAAGAGGC 2716
                              i   v      
C SVA_A#Retropo        449 GGCCGGGCAGAGGC 436

Matrix = 20p53g.matrix
Kimura (with divCpGMod) = 10.39
Transitions / transversions = 3.82 (42/11)
Gap_init rate = 0.08 (36 / 445), avg. gap size = 1.31 (47 / 36)

 1879  13.17 5.91 3.64  UnnamedSequence   2372  2828    379 C SVA_A    Retroposon/SVA    404    870    517    
1879 13.17 5.91 3.64 UnnamedSequence 2372 2828 (379) C SVA_A#Retroposon/SVA (517) 870 404 m_b1s502i20

  UnnamedSequen       2372 CCTCCTGGACGGGGTGGCTGCCGGGCGGAGACGCTCCTCACTTCCCAGAT 2421
                                i        i   v       i   iv                ??
C SVA_A#Retropo        870 CCTCCCGGACGGGGCGGCGGCCGGGCAGAGGGGCTCCTCACTTCCCAGNN 821

  UnnamedSequen       2422 GGGGTGGCTGCCGGGCAGAGACGCTCCTCACTTCTCAGACGGGGCAGCT- 2470
                               i ---           i   i      i  i i        i   -
C SVA_A#Retropo        820 GGGGCG---GCCGGGCAGAGGCGCCCCTCACCTCCCGGACGGGGCGGCTG 774

  UnnamedSequen       2471 GCCGGGCGGAGGGGCT-----CCTCA---CTTCTCAGACGGGGTGGTT-G 2511
                                    -      -----  i  --- i  i i       i  i - 
C SVA_A#Retropo        773 GCCGGGCGG-GGGGCTGACCCCCCCACCTCCTCCCGGACGGGGCGGCTGG 725

  UnnamedSequen       2512 CCAGGCAGAGGG----TCTCCTCACTTCTCAGACGGGGC----GGCCGAG 2553
                             i   i i   ----i i  i   i  i i        ----     i 
C SVA_A#Retropo        724 CCGGGCGGGGGGCTGACCCCCCCACCTCCCGGACGGGGCGGCTGGCCGGG 675

  UnnamedSequen       2554 CAGAGACGCTCCTCACCTCCCAGACGGGGTCTCGGCCGGGCAGAGGCGCT 2603
                            i   i          ?            ---         i        
C SVA_A#Retropo        674 CGGAGGCGCTCCTCACNTCCCAGACGGGG---CGGCCGGGCGGAGGCGCT 628

  UnnamedSequen       2604 CCTCACATCCCAGATG--GGGCGGCGGGGCAGAGGCGCTCCCCACATCTC 2651
                                 ?       i --       v               i   v  i 
C SVA_A#Retropo        627 CCTCACNTCCCAGACGANGGGCGGCCGGGCAGAGGCGCTCCTCACTTCCC 578

  UnnamedSequen       2652 AGACGATG-GGCGGCCGGGCAGAGACGCTCCTCACTTCCTAGATGTGATG 2700
                                iv -               i              i   i ---v 
C SVA_A#Retropo        577 AGACGGGGCGGCGGCCGGGCAGAGGCGCTCCTCACTTCCCAGACG---GG 531

  UnnamedSequen       2701 GCGGCTGGGAAGAGGCGCTCCTCACTTCCTAGATGGGAT-GGCGGCCGGG 2749
                                i   v               ?   i   i--   -          
C SVA_A#Retropo        530 GCGGCCGGGCAGAGGCGCTCCTCACNTCCCAGAC--GATGGGCGGCCGGG 483

  UnnamedSequen       2750 CGGAGACGCTCCTCACTTTCCAGACTG---GGCAGCCAGGCAGAGGGGCT 2796
                            i                i      v ---   i   i        ----
C SVA_A#Retropo        482 CAGAGACGCTCCTCACTTCCCAGACGGGGTGGCGGCCGGGCAGAGG---- 437

  UnnamedSequen       2797 CCTCACATCCCAG-ACGATGGGCGGCC-AGGCAG 2828
                            iv  -    i  -  vv    v    -      
C SVA_A#Retropo        436 CTGCA-ATCCTAGCACTTTGGGAGGCCAAGGCAG 404

Matrix = 20p53g.matrix
Kimura (with divCpGMod) = 10.41
Transitions / transversions = 3.83 (46/12)
Gap_init rate = 0.06 (29 / 456), avg. gap size = 1.52 (44 / 29)

 2350  11.23 4.73 2.31  UnnamedSequence   2450  2914    293 C SVA_B    Retroposon/SVA    392    867    516    
2350 11.23 4.73 2.31 UnnamedSequence 2450 2914 (293) C SVA_B#Retroposon/SVA (516) 867 392 m_b1s502i21

  UnnamedSequen       2450 CACTTCTCAGACGGGGCAGCTGCCGGGCGGAGGGGCTCCTCACTTCTCAG 2499
                              i  i         ii  v       i                 i   
C SVA_B#Retropo        867 CACCTCCCAGACGGGGTGGCGGCCGGGCAGAGGGGCTCCTCACTTCCCAG 818

  UnnamedSequen       2500 ACGGGGTGGTTGCCAGGCAGAGGGTCTCCTCACTTCTCAGACGGGGCGGC 2549
                                 i  ---  i        vv i  i   i  i i           
C SVA_B#Retropo        817 ACGGGGCGG---CCGGGCAGAGGCGCCCCCCACCTCCCGGACGGGGCGGC 771

  UnnamedSequen       2550 ----CGAGCAGAGA----CGCTCCTCACCTCC---CAGACGGGGT--CTC 2586
                           ----  i  i i i----?? i  i       --- i       i--  -
C SVA_B#Retropo        770 TGGCCGGGCGGGGGGCTGNNCCCCCCACCTCCTCCCGGACGGGGCGGCT- 722

  UnnamedSequen       2587 GGCCGGGCAGAGG--CG--CTCCTCACATCCCAGATGGGGCGGCGGG--- 2629
                                   i i  --i --        v       i        v  ---
C SVA_B#Retropo        721 GGCCGGGCGGGGGGCTGACCTCCTCACCTCCCAGACGGGGCGGCCGGCCG 672

  UnnamedSequen       2630 -GCAGAGGCGCTCCCCACATCTCAGACGATGGGCGGCCGGGCAGAGACGC 2678
                           -             i   ?  i      --       v        i   
C SVA_B#Retropo        671 GGCAGAGGCGCTCCTCACNTCCCAGACG--GGGCGGCGGGGCAGAGGCGC 624

  UnnamedSequen       2679 TCCTCACTTCCTAGATGTGATGG-CGGCTGGGAAGAGGCGCTCCTCACTT 2727
                              i   v   i   i --    -    i   v    i            
C SVA_B#Retropo        623 TCCCCACATCCCAGACG--ATGGGCGGCCGGGCAGAGACGCTCCTCACTT 576

  UnnamedSequen       2728 CCTAGATGGGATGGCGGCCGGGCGGAGACGCTCCTCACTTTCCAGACTGG 2777
                             i   i               vi   i            i         
C SVA_B#Retropo        575 CCCAGACGGGATGGCGGCCGGGAAGAGGCGCTCCTCACTTCCCAGACTGG 526

  UnnamedSequen       2778 GCAGCCAGGCAGAGGGGCTCCTCACATCCCAGACGATGGGCGGCCAGGCA 2827
                             i   i                                           
C SVA_B#Retropo        525 GCGGCCGGGCAGAGGGGCTCCTCACATCCCAGACGATGGGCGGCCAGGCA 476

  UnnamedSequen       2828 GAAGAGACACTCCTCACTTCCCAGACGGGGTGGCAGCCGGGCAGAGGCTG 2877
                             ---   i                         i               
C SVA_B#Retropo        475 GA---GACGCTCCTCACTTCCCAGACGGGGTGGCGGCCGGGCAGAGGCTG 429

  UnnamedSequen       2878 CAATCTCGGCACTTTGGGAGGCCAAGGCAGGCGGCTG 2914
                                                                
C SVA_B#Retropo        428 CAATCTCGGCACTTTGGGAGGCCAAGGCAGGCGGCTG 392

Matrix = 20p53g.matrix
Kimura (with divCpGMod) = 8.93
Transitions / transversions = 4.67 (42/9)
Gap_init rate = 0.04 (20 / 464), avg. gap size = 1.65 (33 / 20)
```

```
+   17   12.0  0.0  7.7  UnnamedSequence   3040  3085  (122) + (GGC)n   Simple_repeat       1     42    (0)   3
```

```
ANNOTATION EVIDENCE:
   17  11.98 0.00 7.69  UnnamedSequence   3040  3081    126 + (GGC)n   Simple_repeat       1     39      0    
17 11.98 0.00 7.69 UnnamedSequence 3040 3081 (126) (GGC)n#Simple_repeat 1 39 (0) m_b1s252i0

  UnnamedSequen       3040 GGCGGTCGGGCGGCGGCGGCTGCGGTCGGTCGCGGCAGCGGC 3081
                                - -            v    -   iv     i     
  (GGC)n#Simple          1 GGCGG-C-GGCGGCGGCGGCGGCGG-CGGCGGCGGCGGCGGC 39

Matrix = Unknown
Transitions / transversions = 1.00 (2/2)
Gap_init rate = 0.07 (3 / 41), avg. gap size = 1.00 (3 / 3)

   14  14.91 5.41 0.00  UnnamedSequence   3049  3085    122 + (GCGGCT)n Simple_repeat       1     39      0    
14 14.91 5.41 0.00 UnnamedSequence 3049 3085 (122) (GCGGCT)n#Simple_repeat 1 39 (0) m_b1s252i1

  UnnamedSequen       3049 GCGGCGGCGGCTGCGG-T-CGGTCGCGGCAGCGGCTCCG 3085
                                v          - -   ii     v      v  
  (GCGGCT)n#Sim          1 GCGGCTGCGGCTGCGGCTGCGGCTGCGGCTGCGGCTGCG 39

Matrix = Unknown
Transitions / transversions = 0.67 (2/3)
Gap_init rate = 0.06 (2 / 36), avg. gap size = 1.00 (2 / 2)
```
