# Supplementary figures and images for: Lynch syndrome caused by a pathogenic SINE-VNTR-Alu (SVA) insertion in MSH2 gene identified by long-read DNA sequencing
Source: Fam Cancer. 2026 Jul 9;25(3):75. doi: 10.1007/s10689-026-00588-7 (PMC13350196; doi:10.1007/s10689-026-00588-7)

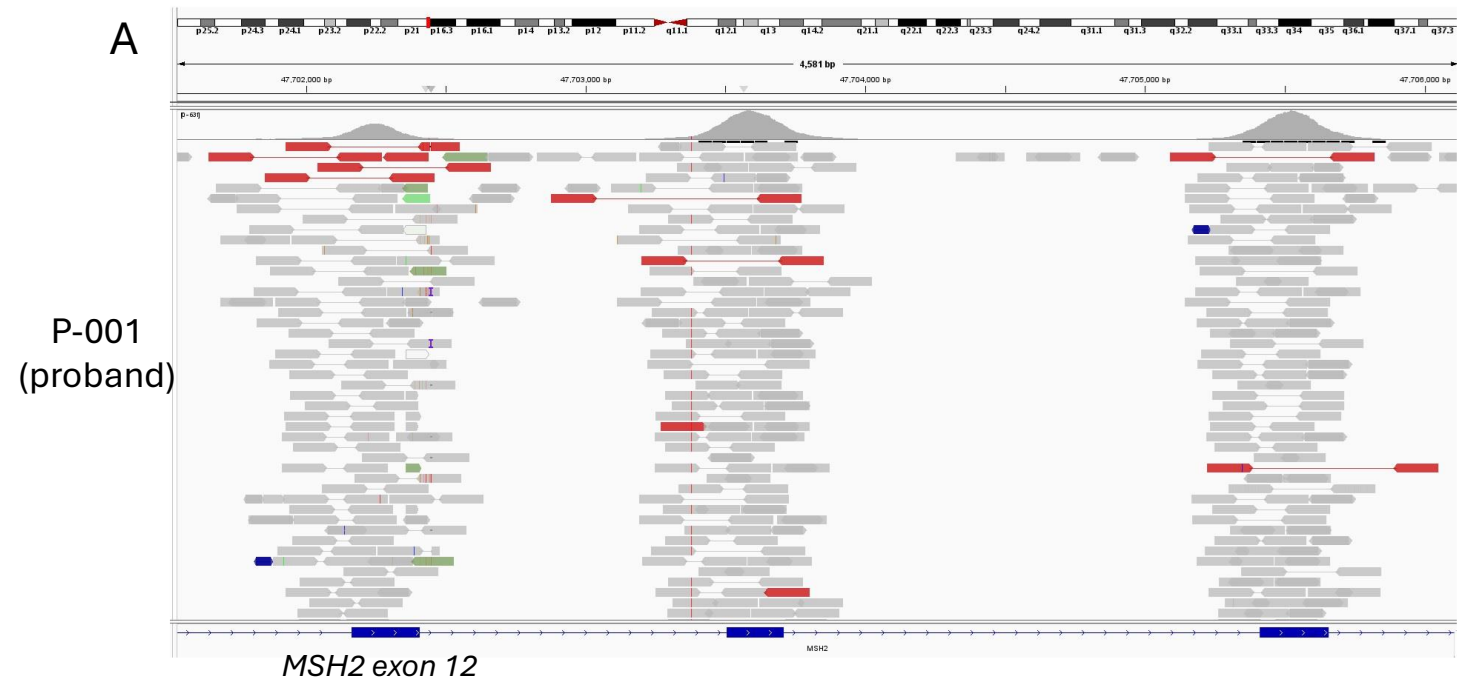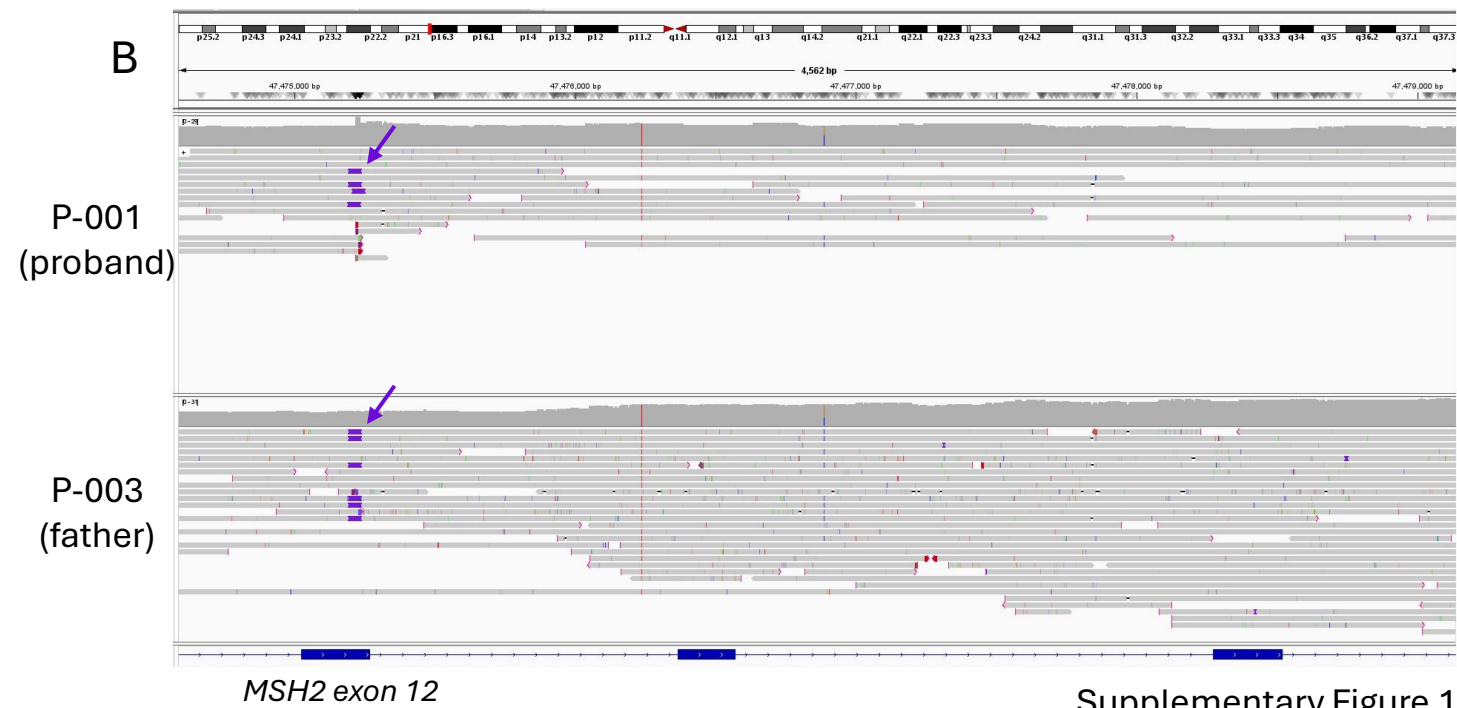

Supplementary Figure 1

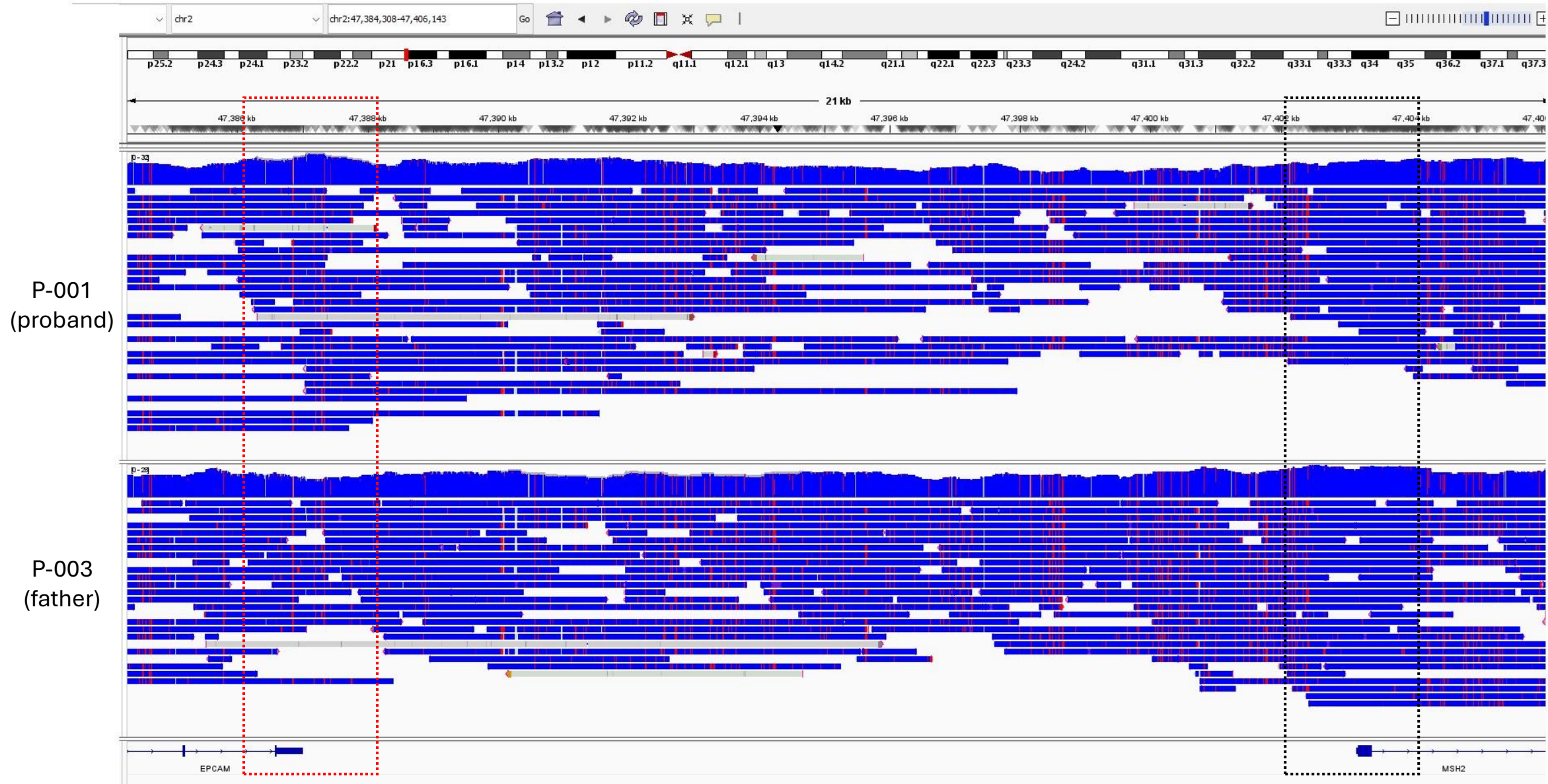

Supplementary Figure 2

Supplement: Supplementary file 2 — Supplementary file2 (PDF 781 KB) [file 10689_2026_588_MOESM2_ESM.pdf]
